# A novel mouse model of Warburg Micro syndrome reveals roles for RAB18 in eye development and organisation of the neuronal cytoskeleton

**DOI:** 10.1242/dmm.015222

**Published:** 2014-04-24

**Authors:** Sarah M. Carpanini, Lisa McKie, Derek Thomson, Ann K. Wright, Sarah L. Gordon, Sarah L. Roche, Mark T. Handley, Harris Morrison, David Brownstein, Thomas M. Wishart, Michael A. Cousin, Thomas H. Gillingwater, Irene A. Aligianis, Ian J. Jackson

**Affiliations:** 1 MRC Human Genetics Unit, Institute for Genetics and Molecular Medicine, University of Edinburgh, Edinburgh EH4 2XU, UK.; 2 The Roslin Institute, University of Edinburgh, Edinburgh EH25 9RG, UK.; 3 Euan MacDonald Centre for Motor Neurone Disease Research and Centre for Integrative Physiology, University of Edinburgh, Edinburgh EH8 9XD, UK.; 4 Centre for Integrative Physiology, University of Edinburgh, Edinburgh EH8 9XD, UK.; 5 Queen’s Medical Research Institute, University of Edinburgh, Edinburgh, EH16 4TJ, UK.

**Keywords:** Warburg Micro syndrome, Cataract, Neurofilament

## Abstract

Mutations in *RAB18* have been shown to cause the heterogeneous autosomal recessive disorder Warburg Micro syndrome (WARBM). Individuals with WARBM present with a range of clinical symptoms, including ocular and neurological abnormalities. However, the underlying cellular and molecular pathogenesis of the disorder remains unclear, largely owing to the lack of any robust animal models that phenocopy both the ocular and neurological features of the disease. We report here the generation and characterisation of a novel *Rab18*-mutant mouse model of WARBM. *Rab18*-mutant mice are viable and fertile. They present with congenital nuclear cataracts and atonic pupils, recapitulating the characteristic ocular features that are associated with WARBM. Additionally, *Rab18*-mutant cells exhibit an increase in lipid droplet size following treatment with oleic acid. Lipid droplet abnormalities are a characteristic feature of cells taken from WARBM individuals, as well as cells taken from individuals with other neurodegenerative conditions. Neurological dysfunction is also apparent in *Rab18*-mutant mice, including progressive weakness of the hind limbs. We show that the neurological defects are, most likely, not caused by gross perturbations in synaptic vesicle recycling in the central or peripheral nervous system. Rather, loss of *Rab18* is associated with widespread disruption of the neuronal cytoskeleton, including abnormal accumulations of neurofilament and microtubule proteins in synaptic terminals, and gross disorganisation of the cytoskeleton in peripheral nerves. Global proteomic profiling of peripheral nerves in *Rab18*-mutant mice reveals significant alterations in several core molecular pathways that regulate cytoskeletal dynamics in neurons. The apparent similarities between the WARBM phenotype and the phenotype that we describe here indicate that the *Rab18*-mutant mouse provides an important platform for investigation of the disease pathogenesis and therapeutic interventions.

## INTRODUCTION

Warburg Micro syndrome (WARBM) is a heterogenous autosomal recessive disorder (Warburg et al., 1993). Loss-of-function mutations have been identified in *RAB3GAP1* (Aligianis et al., 2005), *RAB3GAP2* (Borck et al., 2011), *RAB18* (Bem et al., 2011) and *TBC1D20* (Liegel et al., 2013), and these mutations result in clinically indistinguishable phenotypes. The clinical features of WARBM are primarily ocular and neurological (Abdel-Salam et al., 2007; Derbent et al., 2004; Handley et al., 2013): affected children have visual impairment and eye abnormalities – including congenital bilateral cataracts, microphthalmia, microcornea (<10 mm diameter) and small atonic pupils that do not react to dark or mydriatic agents. Despite early cataract surgery, WARBM individuals can only perceive light and are effectively blind, as a result of progressive optic nerve atrophy, with a normal electroretinogram but absent visually evoked potentials. WARBM individuals are severely neurologically handicapped. Characteristically, the affected individuals show congenital truncal hypotonia and, from ~8–12 months, have lower-limb spasticity, which is progressive and eventually affects the upper limbs, leading to spastic quadriplegia later in life. Nerve conduction studies show evidence of a progressive axonal peripheral neuropathy. Affected children, additionally, have hypothalamic hypogonadism, postnatal growth retardation and global developmental delay.

RAB proteins function as molecular switches, cycling between ‘inactive’ GDP-bound and ‘active’ GTP-bound conformations in order to regulate membrane trafficking in a spatially and temporally restricted manner. RAB protein cycling is governed by four classes of protein – RAB GDP dissociation inhibitor (GDI), RAB guanine nucleotide exchange factor (GEF), RAB GDP displacement factor (GDF) and RAB GTPase activating protein (GAP) (Corbeel and Freson, 2008; Goody et al., 2005; Stenmark, 2009). RAB3GAP1 (catalytic subunit) and RAB3GAP2 (noncatalytic subunit) form a heterodimeric enzyme complex, which has GAP activity that is specific for the RAB3 family of proteins, hydrolysing GTP into GDP and regulating the Ca^2+^-mediated exocytosis of hormones and neurotransmitters (Aligianis et al., 2005; Südhof, 2004). TBC1D20 is a GAP that specifically acts on the RAB1- and RAB2-family proteins in COPII-dependent endoplasmic reticulum to Golgi transport (Haas et al., 2007; Nevo-Yassaf et al., 2012). However, the role(s) of RAB18 in trafficking are still emerging – RAB18 has been reported to localise to lipid droplets in adipocytes, fibroblasts and epithelial cells, where it has a role in lipolysis and lipogenesis (Martin et al., 2005; Ozeki et al., 2005; Pulido et al., 2011). Localisation to the endoplasmic reticulum and, in variable degrees, to the Golgi apparatus has also been described (Dejgaard et al., 2008). In neuroendocrine cells, RAB18 has been reported to regulate Ca^2+^-mediated exocytosis (Vazquez-Martinez et al., 2007). Taken together, these data suggest that RAB18 has discrete cellular roles in different cell types (Martin et al., 2005; Ozeki et al., 2005), but these studies provide no clue as to its role in WARBM disease pathogenesis.

**TRANSLATIONAL IMPACT**

**Clinical issue**

Loss-of-function mutations in *RAB18*, which encodes a ubiquitously expressed Rab-associated protein, cause the autosomal recessive disorder Warburg Micro syndrome. Individuals with this disorder present with abnormalities in the ocular, neurological and endocrine systems, in addition to postnatal growth retardation. However, little progress has been made in elucidating the underlying pathology as the families of affected individuals often decline postmortem examination. Furthermore, although animal models with mutations in other implicated genes have been described, none of these have recapitulated both the ocular and neurological features of the human disease. The effects of knocking out *RAB18* have not been explored in an animal model to date.

**Results**

In this study, the authors generated and characterised a knockout mouse model for *Rab18*. They report that homozygous *Rab18* mice display many of the major ocular and neurological abnormalities that are associated with Warburg Micro syndrome, including congenital nuclear cataracts, atonic (constricted) pupils and progressive limb weakness. The group show that loss of *Rab18* is associated with widespread disruption of the neuronal cytoskeleton – including abnormal accumulations of neurofilament and microtubule proteins in synaptic terminals – and gross disorganisation of the cytoskeleton in peripheral nerves. Global proteomic profiling of peripheral nerves in *Rab18*-mutant mice revealed significant alterations in several core molecular pathways that regulate cytoskeletal dynamics in neurons.

**Implications and future directions**

This work provides the first animal model that recapitulates the ocular and neurological abnormalities that are observed in individuals with Warburg Micro syndrome. The study highlights an important role for *Rab18* in eye development and a previously unknown role in the maintenance of cytoskeletal organisation in the peripheral nervous system. The data suggest that the *Rab18*-knockout mouse represents a robust model for Warburg Micro syndrome, offering a new experimental platform for investigating disease pathogenesis and testing potential therapies.

*Rab3gap1* mutant mice and knockout mice (targeting two, three or four members of the *Rab3* subfamily) have previously been generated (Sakane et al., 2006; Schlüter et al., 2004). None of these lines recapitulated the major clinical features of WARBM (Sakane et al., 2006; Schlüter et al., 2004). *Rab3gap1* mutant mice are viable, fertile and live a normal lifespan (Sakane et al., 2006). They show altered synaptic transmission *in vitro*, inhibition of Ca^2+^-mediated exocytosis of glutamate from cortical synaptosomes and accumulation of GTP-bound RAB3A in the brain, but only mild alterations in short-term plasticity in the hippocampal CA1 region. They show no neurological deterioration and no structural eye, brain or genital abnormalities (Sakane et al., 2006). Recently, mutations in *Tbc1d20* have been reported in the *blind sterile* (*bs*) mouse (Liegel et al., 2013). These mice exhibit nuclear cataracts from postnatal day (P)14 and male infertility (Liegel et al., 2013; Varnum, 1983) but do not show any neurological phenotype. No mammalian models for *Rab18* deficiency have been reported, although morpholino knockdown of the two *RAB18* orthologues in zebrafish results in animals with reduced body size, microphthalmia and microcephaly, reminiscent of the WARBM phenotype (Bem et al., 2011). Animal models recapitulating both ocular and neurological phenotypes of WARBM are required in order to investigate the ultrastructural and molecular aspects of disease pathogenesis that cannot be identified through examination of the affected individuals.

Here, we report the generation and characterisation of a novel *Rab18*-mutant mouse model of WARBM. *Rab18*-mutant mice develop the ocular and neurological phenotypes that are associated with the human disease, including congenital nuclear cataracts, atonic pupils and progressive hind limb weakness. The treatment of cells, derived from the mouse model, to induce lipid droplet formation results in abnormally large lipid droplets, emphasising a link between lipid droplets and neurological disease. We show that the neurological defects in *Rab18*-mutant mice are not caused by gross abnormalities in synaptic vesicle recycling, but rather that they are associated with accumulations of neurofilament and microtubules in synaptic terminals, and cytoskeletal disorganisation in the sciatic nerve. Using global proteomic profiling of peripheral nerves, we reveal alterations in several key pathways that are involved in the organisation and maintenance of cytoskeletal architecture in *Rab18^−/−^* sciatic nerve.

## RESULTS

### *Rab18^−/−^* mice recapitulate the Warburg Micro syndrome phenotype

**Fig. 1.**
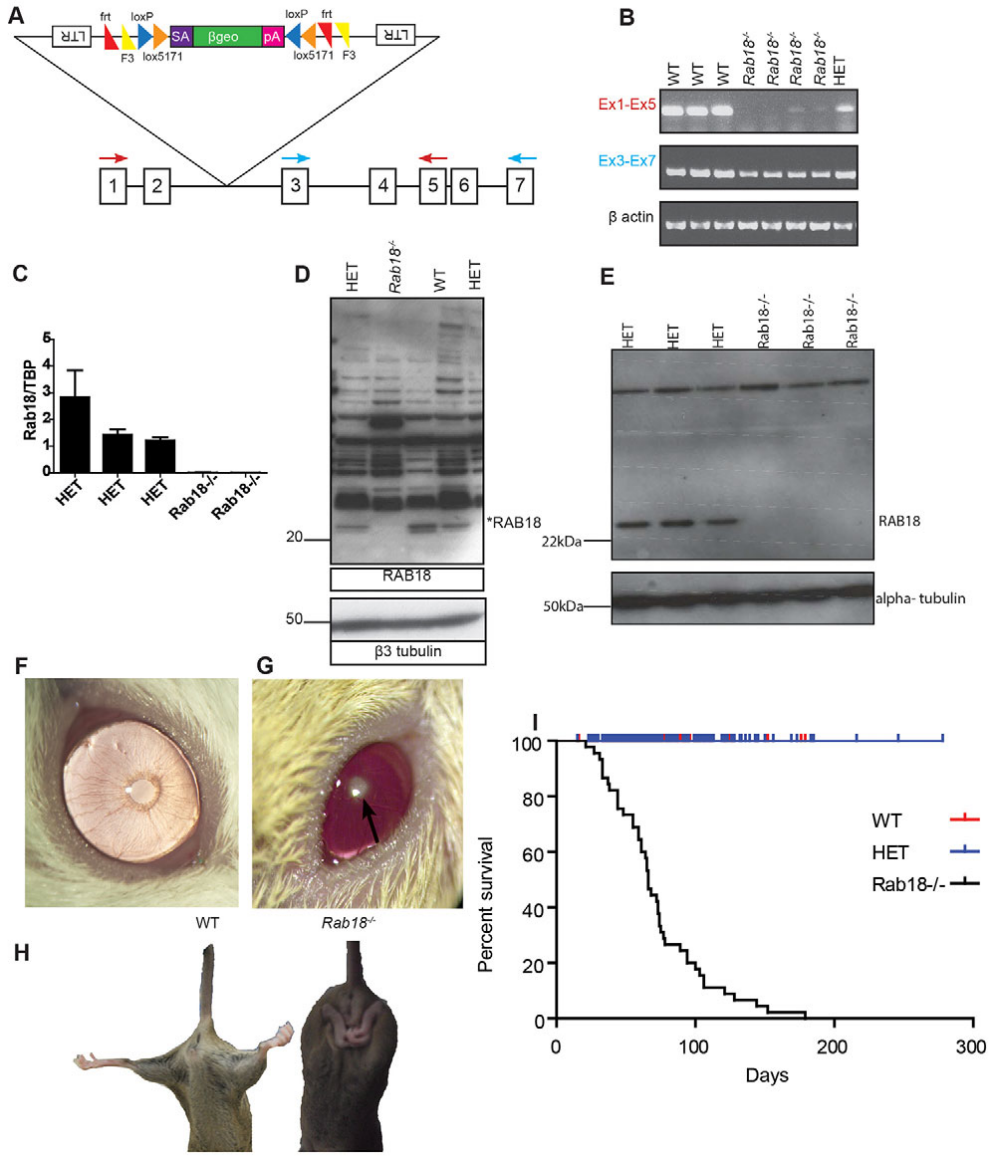
***Rab18^−/−^* mice recapitulate the Warburg Micro syndrome phenotype.** (A) Representation of the FlipRosaβGeo genetrap cassette inserted into intron 2 of *Rab18*. LTR, long terminal repeat; frt and F3, target for FLPe recombinase; loxP and lox5171, targets for Cre recombinase; SA, splice acceptor; βgeo, β-galactosidase and neomycin phosphotransferase fusion gene as a marker of protein expression; pA, polyadenylation sequence, adapted from Schnütgen et al., 2005. (B) Reverse-transcriptase PCR on cDNA taken from whole embryos of wild-type (WT), heterozygote (HET) and *Rab18^−/−^* mice. The products were amplified using oligonucleotides that targeted exons 1 and 5 (red arrows in A) and showed weak residual *Rab18* transcript in *Rab18^−/−^* mice. Amplification using oligonucleotides that targeted exons 3 and 7 (blue arrows in A) showed slightly reduced transcript levels from the 3′-end of the cassette insertion site in *Rab18^−/−^* mice. β-actin was used as a reaction control. (C) Quantitative RT-PCR on heterozygous (HET) and *Rab18^−/−^* mouse embryonic fibroblasts that amplified exons 3–4 showed no *Rab18* transcript in *Rab18^−/−^* mice compared with heterozygous littermate controls. The relative expression of *Rab18* to the expression of *TBP* is shown. (D) Representative western blot analysis on 10 μg of sciatic nerve isolated from wild-type (WT), heterozygous (HET) and *Rab18^−/−^* mice using an antibody targeting the C-terminus of RAB18. The western blot shows a lack of RAB18 protein in *Rab18^−/−^* mice. β3 tubulin was used as a loading control. (E) Representative western blot analysis of heterozygous (HET) and *Rab18^−/−^* mouse embryonic fibroblasts. RAB18 protein expression was lacking in *Rab18^−/−^* mouse embryonic fibroblasts, α tubulin was used as a loading control. (F) Slit-lamp picture of the eye of a control mouse. (G) Slit-lamp pictures showed dense cataracts in adult *Rab18^−/−^* mice. (H) Representative images of hind limb clasping in *Rab18^−/−^* mice. When elevated by the tail, wild-type mice (WT, left) spread their hind limbs, whereas their *Rab18^−/−^* littermates (right) clasped their hind paws together. (I) Kaplan-Meirer plot showing the survival of wild-type (WT), heterozygote (HET) and *Rab18^−/−^* mice. *Rab18^−/−^* mice were culled following onset of hind limb weakness in accordance with Home Office guidelines.

Our initial aim was to create a mouse model of Warburg Micro syndrome. *Rab18*-mutant mice were generated from embryonic stem cells with the *Rab18*^Gt(EUCE0233a03)Hmgu^ allele (hereafter referred to as *Rab18^−^*), which contains the genetrap vector inserted into intron 2 of *Rab18* (Fig. 1A). The genetrap allele initiates transcription from the endogenous promoter and prematurely terminates at the polyadenylation sequence in the FlipRosaβGeo vector, resulting in a truncated mRNA that encodes a non-functional protein. To evaluate the impact of the genetrap insertion on *Rab18* mRNA expression, reverse transcriptase PCR was performed on cDNA that had been extracted from embryonic day (E)11.5 embryos. Primers flanking the genetrap insertion site showed very weak *Rab18* expression in *Rab18^−/−^* embryos (exons 1–5, Fig. 1B, top panel). Primers further towards the 3′-end of the cassette (exons 3–7) amplified products in all genotypes, albeit at a lower intensity in *Rab18^−/−^* embryos, indicating alternate transcripts, which could potentially produce alternate protein isoforms (Fig. 1B, middle panel). However, quantitative real-time (RT)-PCR that amplified exons 4–5 in mouse embryonic fibroblasts (MEFs) (Fig. 1C) showed that any alternate transcripts were present at very low levels, and western blot analysis of sciatic nerves (Fig. 1D) and mouse embryonic fibroblasts (Fig. 1E), by using an antibody that targeted the C-terminus of RAB18, identified no residual RAB18 expression in *Rab18^−/−^* mice, nor any smaller protein isoform. We, therefore, consider *Rab18^−/−^* mice to represent the null phenotype of *Rab18*.

*Rab18^−/−^* mice were viable and fertile. However, both heterozygote and *Rab18^−/−^* mice were found at non-Mendelian ratios at weaning, but not embryonic stages, suggesting some perinatal death (supplementary material Table S1). At the time of eye opening (postnatal day 12), *Rab18^−/−^* mice were readily identifiable by the presence, with complete penetrance, of dense nuclear cataracts (Fig. 1G), which were absent in the eyes of control mice (Fig. 1F), and atonic pupils, recapitulating the characteristic ocular phenotypes that are associated with WARBM. From three weeks of age, when elevated by the tail, all *Rab18^−/−^* mice showed abnormal hind limb grasping (Fig. 1H, right) compared with control animals that spread their hind limbs (Fig. 1H, left), indicative of neurological dysfunction. This abnormal hind limb phenotype progressed at variable ages to splayed and weak hind limbs. All mice reaching this stage were culled in accordance with Home Office guidelines (Fig. 1I). Heterozygote mice were indistinguishable from their wild-type littermates. Detailed necropsy analysis was performed on two symptomatic *Rab18^−/−^* mice and littermate controls. The tissues examined were heart, lung, thyroid, adrenals, testis or uterus, stomach, small intestine, caecum, colon, mesentery, liver, skin, abdominal mammary, brain (rostral to the optic chiasm, at the caudal borders of the mammary bodies to expose the thalamus and caudal section of the hippocampus, and at the widest point of the cerebellum), pituitary, lower urinary, kidneys, interscapular brown fat, spleen, pancreas, thymus, lymphosalivary, head, lumbar and thoracic, and cervical spine and a cross-section of thigh muscles. No substantial differences were found, suggesting that the overt phenotypes that were observed in *Rab18^−/−^* mice were not caused by gross disruption of normal development that resulted from global loss of *Rab18* expression but, rather, represented specific pathological responses in a restricted range of cells and tissues.

### Characterisation of ocular defects in *Rab18^−/−^* mice

**Fig. 2.**
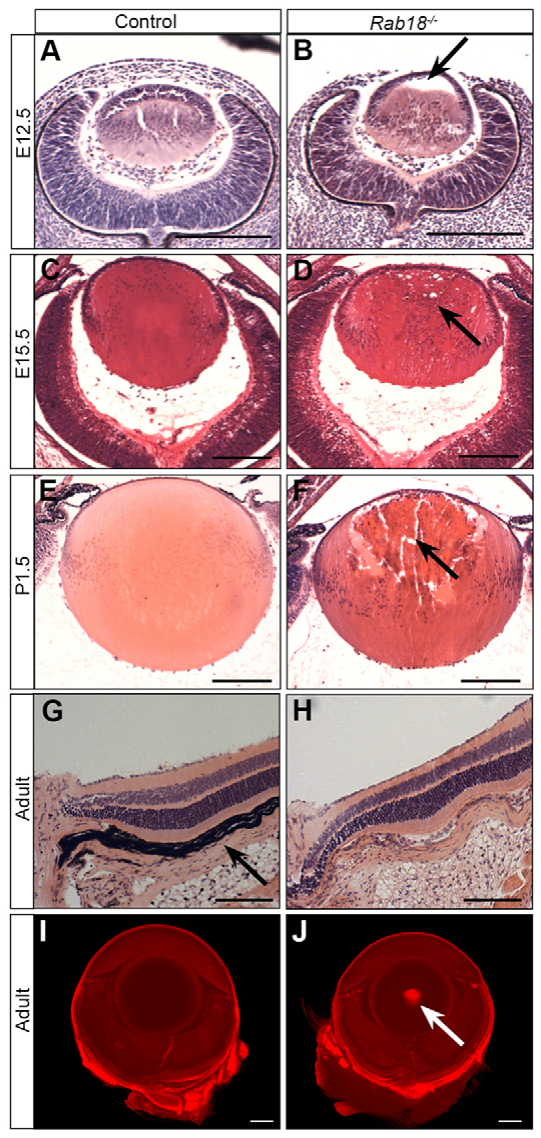
**Defective pre-natal development of the lens in *Rab18^−/−^* mice.** (A,B) At E12.5, *Rab18^−/−^* mice showed a delay in the filling of the lens vesicle through the delayed migration of lens fibre cells from the posterior (B, arrow), whereas wild-type littermates had a closed lens vesicle (A). By E15.5, the *Rab18^−/−^* lens vesicle had closed, but the first signs of cataract development (small vacuoles at the lens periphery) were evident (D, arrow). Signs of cataract formation were absent in controls (C). By P1.5, large vacuoles and centralised pyknotic nuclei were seen in *Rab18^−/−^* lenses (F, arrow) but not in controls (E). All other ocular structures in control (G) and *Rab18^−/−^* lenses (H) appeared normal, and no retinal degeneration was observed. Note that the *Rab18^−/−^* eye was unpigmented, owing to the origin of the mutation in 129P2-derived embryonic stem cells; consequently, the retinal pigment epithelium (arrow in G) was not easily observed. Optical projection tomography undertaken on adult wild-type (I) and *Rab18^−/−^* unpigmented eyes (J) showed a dense nuclear cataract in the centre of the *Rab18^−/−^* lens (arrow in J). Scale bars: 100 μm (A–F); 50 μm (G,H); 400 μm (I,J).

To further our characterisation of the ocular defects that were observed in our initial screen of *Rab18^−/−^* mice, we performed histological analysis of haematoxylin- and eosin-stained control and *Rab18^−/−^* eyes at various stages of embryogenesis (Fig. 2). During embryogenesis, the lens forms from an invagination of the lens placode, producing a hollow ball of epithelial cells. Cells lining the posterior of the vesicle then elongate to the anterior, closing the vesicle by E12.5 (Fig. 2A). At E12.5 in *Rab18^−/−^* mice, the posterior epithelial cells had not reached the lens anterior, suggesting a delay in development (Fig. 2A,B, arrow in B). By E15.5, the mutant lens vesicle had closed, but vacuoles had formed at the periphery (Fig. 2C,D, arrow in D). At P1.5 (Fig. 2E,F), large vacuoles, pyknotic nuclei and nuclear aggregates were evident, all of which are characteristic of cataract development (Fig. 2F, arrow). Individuals with WARBM do not show retinal degeneration and, similarly, examination of the retina in adult *Rab18^−/−^* mice identified no degeneration (Fig. 2G,H). To examine the cataracts in adult mice, optical projection tomography was performed on unpigmented *Rab18^−/−^* mice and littermates. Virtual sections through the three-dimensional reconstruction of the eyes showed a dense nuclear cataract in the mutant eyes (Fig. 2I,J, arrow in J; supplementary material Movies 1, 2). Additionally, individuals with WARBM have permanently constricted pupils, as well as congenital cataracts. Examination of the pupillary response of *Rab18^−/−^* mice to dark, or to the mydriatic agent tropicamide, showed permanently constricted pupils in all mutant eyes. Thus, *Rab18^−/−^* mice recapitulated the core ocular phenotypes that are reported in WARBM individuals, and confirm a key role for RAB18 in eye development.

### Loss of *Rab18* has no effect on synaptic vesicle recycling in the PNS or CNS

Given that loss-of-function mutations in any of the known genes – *RAB3GAP1*, *RAB3GAP2*, *RAB18* or *TBC1D20* – cause a clinically indistinguishable phenotype (Aligianis et al., 2005; Bem et al., 2011; Borck et al., 2011; Liegel et al., 2013), we asked whether the neurological deterioration that was observed in *Rab18^−/−^* mice could be caused by aberrant expression of the RAB3GAP1, RAB3GAP2 or RAB3A proteins. Western blot analysis of isolated synaptic preparations from *Rab18^−/−^*, and control, mouse brains showed no alterations in the levels of RAB3GAP1, RAB3GAP2 or RAB3A (supplementary material Fig. S1A–C), suggesting that any neurological phenotype observed in *Rab18^−/−^* mice was not a consequence of changes in these proteins.

Because *RAB3GAP1* and *RAB3GAP2* were the first genes found to be associated with WARBM, previous studies that attempted to elucidate the pathogenesis underlying WARBM have focused on dysregulation of synaptic vesicle recycling. Therefore, we utilised synaptic vesicle recycling assays in the peripheral (PNS) and central nervous system (CNS) to assess whether loss of RAB18 had any effect on gross parameters of synaptic transmission (supplementary material Fig. S1D–N). For analyses in the PNS (supplementary material Fig. S1D–K), recycling synaptic vesicles at neuromuscular synapses of lumbrical muscles were labelled with the styryl dye FM1-43fx (green, supplementary material Fig. S1E,H) and postsynaptic acetylcholine receptors with tetramethyl-rhodamine isocyanate (TRITC)-conjugated α-bungarotoxin (BTX, red, supplementary material Fig. S1D,G). Following membrane depolarisation with a high potassium solution, all of the nerve terminals that were examined endocytosed the styryl dye to an equal degree, irrespective of genotype (supplementary material Fig. S1J,K). Thus, loss of *Rab18* has no major implications on synaptic vesicle recycling at peripheral synapses.

To examine synaptic vesicle recycling in the CNS (supplementary material Fig. S1L–N), isolated cortical neuron cultures from wild-type and *Rab18^−/−^* mice were transfected with the genetic reporter synaptophysin-pHluorin (sypHy) (Granseth et al., 2006). pHluorins report the pH of their immediate environment. In the case of sypHy, pHluorin is fused to the second intraluminal domain of synaptophysin, such that, upon endocytosis, its fluorescence signal is quenched by the low pH (approximately pH 5.5) of the synaptic vesicle and, upon exocytosis, fluorescence increases as it encounters the extracellular medium. To determine how the absence of RAB18 impacts on the kinetics of endocytosis in the CNS, isolated cortical neuron cultures were stimulated with a train of action potentials (supplementary material Fig. S1L). The change in sypHy fluorescence intensity following stimulation was comparable between wild-type and *Rab18^−/−^* neurons. RAB18 deletion did not affect either sypHy peak response (supplementary material Fig. S1M, percentage of total vesicle pool – wild-type 32.1±3.4%; *Rab18^−/−^* 30.3±4.3%) or the speed of vesicle endocytosis (supplementary material Fig. S1N, time constant for endocytosis – wild-type 42.8±4.7 seconds; *Rab18^−/−^* 39.3±3.3 seconds). We conclude that loss of RAB18 has no effect on synaptic vesicle recycling in either the PNS or CNS and that deficiency in recycling is unlikely to underlie the disease process.

### Enlarged lipid droplets in *Rab18^−/−^* mouse embryonic fibroblasts

**Fig. 3.**
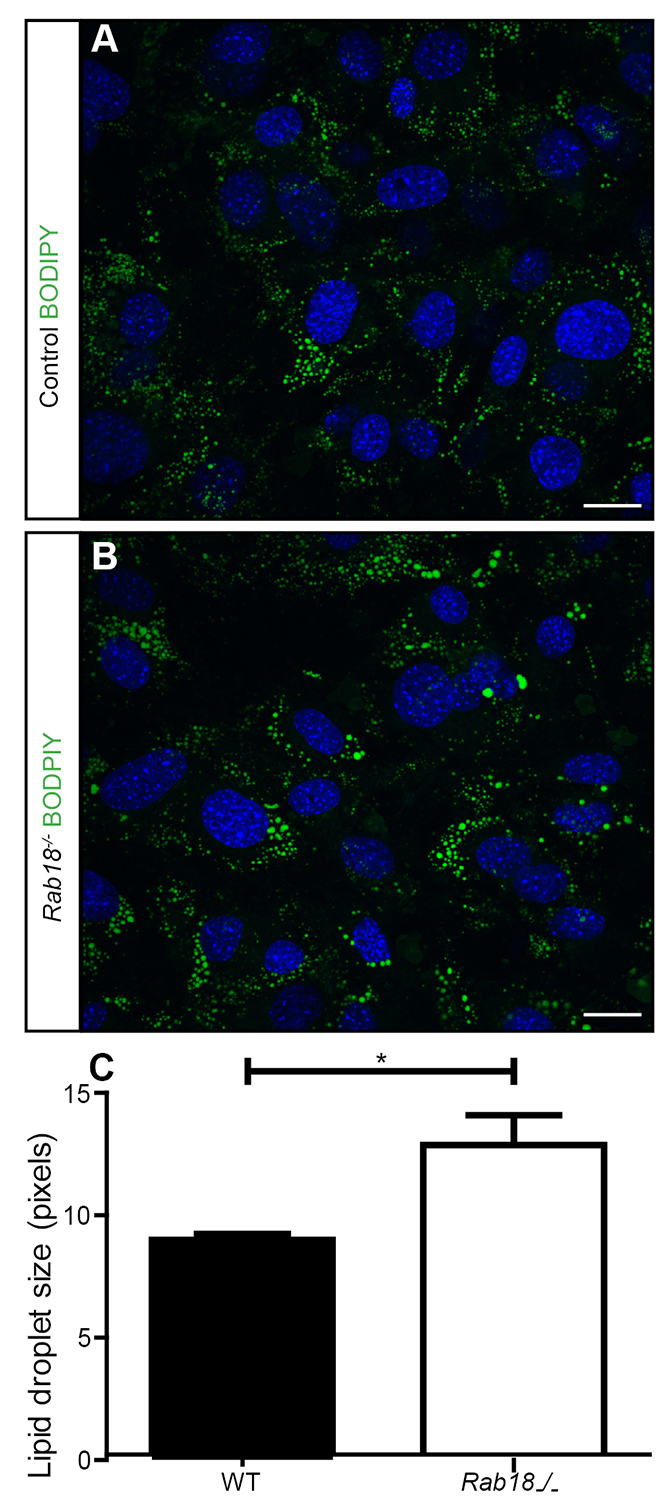
**Loss of RAB18 results in the enlargement of lipid droplets in MEFs.** Control (A) and *Rab18^−/−^* (B) MEFs that had been treated with oleic acid for 24 hours were fixed and stained with the neutral lipid stain BODIPY 493/503 (green), cell nuclei were stained by using DAPI (blue). (C) Quantification of lipid droplet size showed enlarged lipid droplets in *Rab18^−/−^* MEFs. *n*=3 mice, mean±s.e.m. (mean lipid droplet size – 8.9 pixels in control MEFs and 12.8 pixels in Rab18^−/−^ MEFs), *P<0.05 using an unpaired Student’s *t*-test. Scale bars: 10 μm.

RAB18 has previously been reported to localise to lipid droplets in adipocytes, fibroblasts and epithelial cell lines (Martin et al., 2005; Ozeki et al., 2005; Pulido et al., 2011), and has also been identified as a component of lipid droplets in a proteomics screen (Ozeki et al., 2005). Furthermore, enlarged lipid droplet size has also been reported in *bs* MEFs and *TBC1D20*-, *RAB18*- and *RAB3GAP1*-deficient fibroblasts that were taken from WARBM individuals (Liegel et al., 2013). MEFs from control and *Rab18^−/−^* embryos were treated with oleic acid to induce lipid droplet formation and labelled with the neutral lipid stain BODIPY 493/503. After 24 hours of treatment with oleic acid, we observed no difference in the number of lipid droplets, but we did observe an enlargement of lipid droplet size in *Rab18^−/−^* MEFs (mean lipid droplet size – 8.89±0.24 pixels in control and 12.82±1.23 pixels in *Rab18^−/−^*) (Fig. 3), consistent with the phenotype that is observed in *RAB18*-deficient fibroblasts taken from affected individuals.

### Neurofilament accumulation and cytoskeletal disorganisation in the PNS of *Rab18^−/−^* mice

Individuals with WARBM display progressive limb spasticity, leading to spastic quadriplegia, and also peripheral neuropathy. We observed progressive hind limb weakness in our *Rab18^−/−^* mice and, therefore, used the *Rab18^−/−^* mouse model to investigate the underlying causes of neuromuscular defects in WARBM individuals. Initially, we examined whether the observed neuromuscular dysfunction could be associated with atrophy of skeletal muscles. The diameters of individual lumbrical muscle fibres were measured, and no discernible differences were identified at early- or mid-late-symptomatic timepoints (supplementary material Fig. S2A). Additionally, the area of motor endplates from control and *Rab18^−/−^* lumbrical muscles was examined, and again, no difference in morphology or size was observed (supplementary material Fig. S2B–D).

**Fig. 4.**
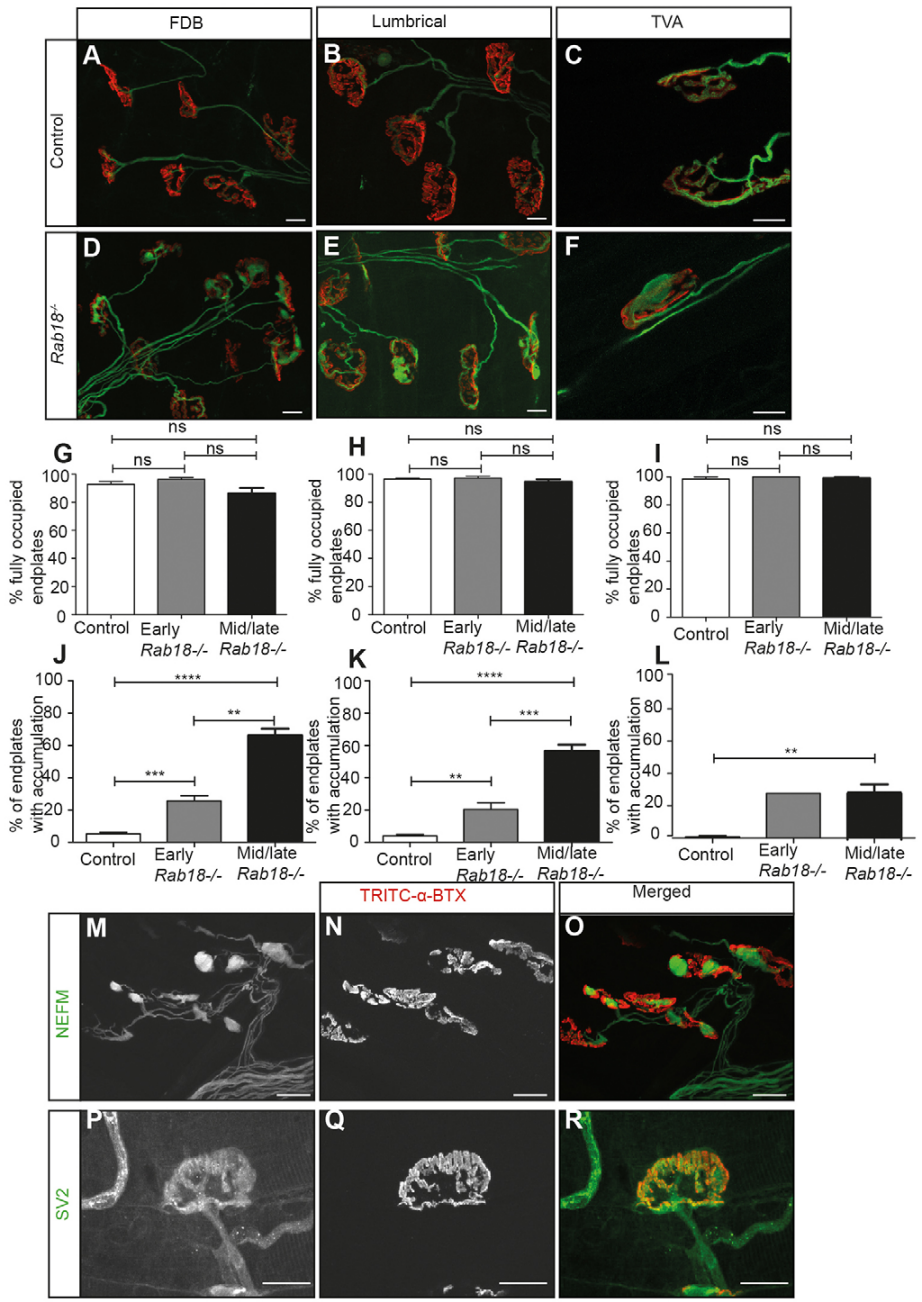
***Rab18^−/−^* mice have large accumulations of neurofilament at the neuromuscular junction.** (A–F) Confocal micrographs showing the neuromuscular junction in (A,D) flexor digitorum brevis (FDB), (B,E) lumbrical and (C,F) transverse abdominus muscle preparations from control (A–C) and *Rab18^−/−^* mice (D–F). TRITC-conjugated α-bungarotoxin staining is shown in red, staining of 165-kDa neurofilament and synaptic vesicle protein SV2 are both shown in green. (G–I) Bar charts (mean±s.e.m.) showing that, in all muscles examined – (G) FDB, (H) lumbrical and (I) TVA – the majority of endplates from both early- and mid-late-symptomatic *Rab18^−/−^* mice were fully occupied (where SV2 staining overlayed the endplate). Statistical significance was assessed using a Mann–Whitney U test. Early symptomatic: FDB *P*-value=0.5553, muscles from control mice (*n*=8) and muscles from Rab18^−/−^ mice (*n*=7); lumbrical *P*-value=0.8120, muscles from control mice (*n*=10) and muscles from Rab18^−/−^ mice (*n*=8); TVA muscle (*n*=1). Mid-late symptomatic: FDB *P*-value=0.3523, muscles from control mice (*n*=9) and muscles Rab18^−/−^ mice (*n*=8); lumbrical *P*-value=1.0, muscles (*n*=10); TVA *P*-value=1.0, muscles (*n*=3). ns, not significant. (J–L) Bar charts (mean±s.e.m.) showing the percentage of endplates that exhibited large accumulations (stained green) in Rab18^−/−^ mice at early- and mid-late-symptomatic timepoints. Statistical significance was assessed using a Mann–Whitney U test. Early symptomatic: FDB *P*-value=0.0026, muscles from control mice (*n*=8) and muscles from Rab18^−/−^ mice (*n*=7); lumbrical *P*-value=0.0096, muscles from control mice (*n*=10) and muscles from Rab18^−/−^ mice (*n*=8); TVA muscle (*n*=1). Mid-late symptomatic: FDB *P*-value=0.0005, muscles from control mice (*n*=9) and muscles from Rab18^−/−^ mice (*n*=8); lumbrical *P*-value=0.0002, muscles (*n*=10); TVA *P*-value=0.0765, muscles (*n*=3). ns, not significant, **P<0.005, ***P<0.001. (M–R) Confocal micrographs showing neuromuscular junctions from Rab18^−/−^ lumbrical muscles that had been immunostained with TRITC-conjugated α-bungarotoxin (red) and one of either neurofilament (NEFM) (M–O) or SV2 (P–R). Note the large neurofilament accumulations in Rab18^−/−^ endplates (O) but normal levels of synaptic vesicle marker SV2 (R). Scale bars: 10 μm.

Because the hind limb phenotype of *Rab18^−/−^* mice was not associated with gross pathological changes in muscle, we proceeded to examine the integrity of nerve-muscle connectivity at the neuromuscular junction (NMJ). The NMJ was examined in the flexor digitorum brevis (FDB) and lumbrical muscles from the hind paw, and the transverse abdominus (TVA) muscle from the anterior abdominal wall in early (3-weeks old, onset of hindlimb clasping phenotype) and mid-late-symptomatic (splayed and weak hind limbs) *Rab18^−/−^* mice, and littermate controls. Postsynaptic motor endplates (labelled with TRITC-conjugated α-bungarotoxin) conformed to the normal ‘pretzel’ shape and were normally innervated in all of the muscle types that were examined (165-kDa neurofilament and the synaptic vesicle protein SV2) (Fig. 4A–I). This suggests that the neuromuscular junctions developed normally. Additionally, no hallmark features of ‘dying-back’ pathology (such as partial or vacant endplates) were observed in *Rab18^−/−^* mice as hind limb weakness progressed. However, during our analysis of motor endplate occupancy, we observed that the vast majority of motor nerve terminals of distal motor axons were grossly abnormal. NMJs in all three muscle types from *Rab18^−/−^* mice showed distinctive large accumulations at both stages, progressing from 30% of terminals in early symptomatic muscles to 60% by mid-late-symptomatic timepoints in FDB and lumbrical muscles (Fig. 4J–K). The postural TVA muscle also showed abnormal accumulations at ~20% of motor nerve terminals in both early- and mid-late-symptomatic timepoints (Fig. 4L). Thus, the phenotype was more severe in those muscles that demonstrated weakness. To determine the nature of the accumulations, separate immunostaining of motor nerve terminals and distal axons in lumbrical muscles from mid-late symptomatic *Rab18^−/−^* mice was performed by using antibodies against one of either 165-kDa neurofilament (Fig. 4M–O) or SV2 (Fig. 4P–R). This showed that the abnormal accumulations contained neurofilament proteins.

**Fig. 5.**
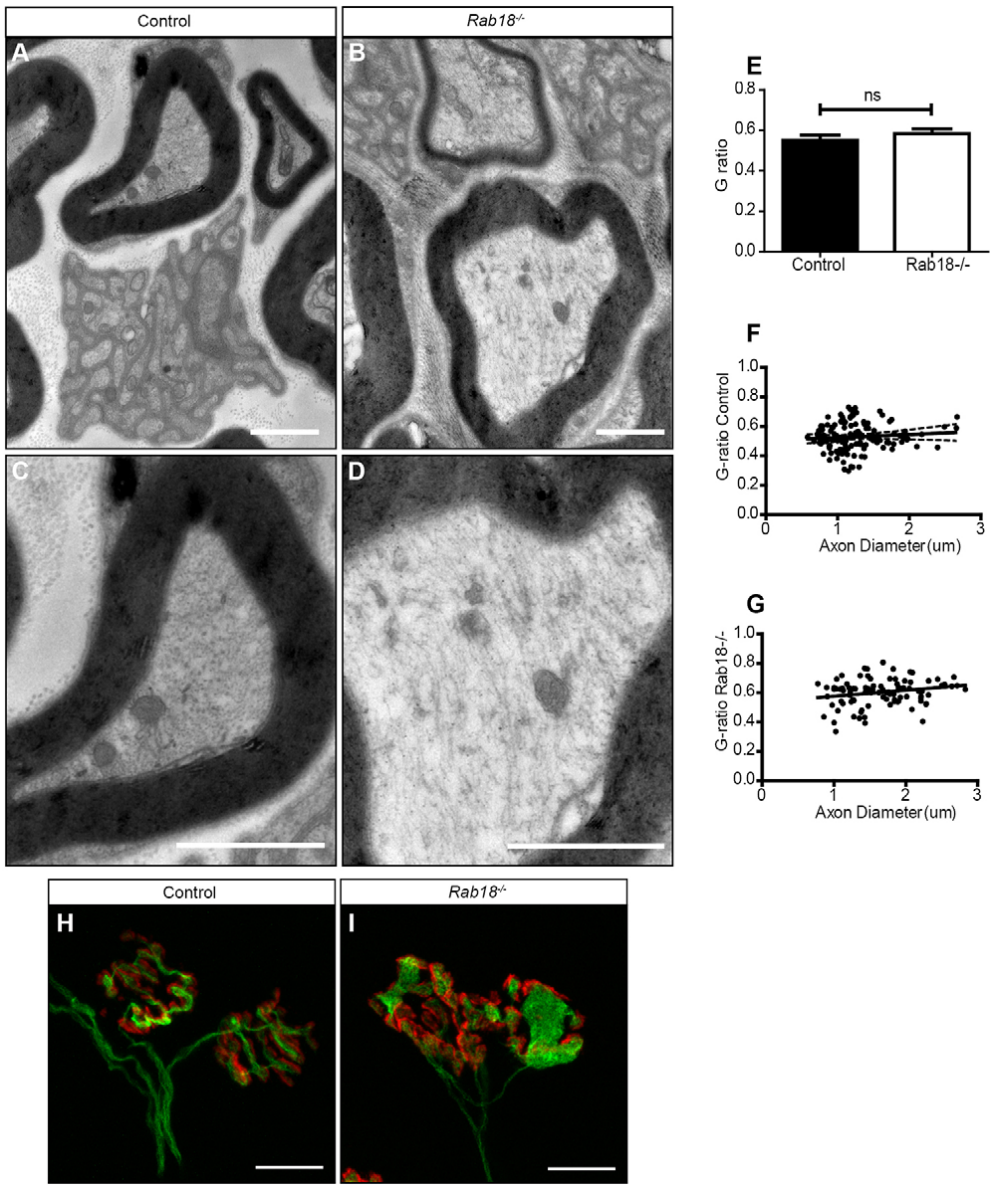
***Rab18^−/−^* mice have grossly disorganised cytoskeletons in peripheral nerves and accumulate microtubules at the neuromuscular junction.** (A–D) Electron micrographs of the sciatic nerve in control (A,C) and mid-late-symptomatic *Rab18^−/−^* mice (B,D) showed normal myelination and normal Remak bundles in both genotypes. (C,D) Higher-magnification images showed disorganisation of the cytoskeleton in the sciatic nerve of *Rab18^−/−^* mice (D) compared with that of controls (C). The images are representative of that found in three animals for each genotype. (E–G) Quantification of myelination identified no abnormalities in the G-ratio (G-ratio is the diameter of an axon divided by the diameter of axon plus myelin). Statistical significance was tested by using an unpaired two-tailed Student’s *t*-test. G-ratio *P*-value=0.7610, mice (*n*=3) (E) or spread of axon diameter compared with the G-ratio in control (F) and *Rab18*^−/−^ mice (G). (H,I) FDB muscles from wild-type (H) and mid-late-symptomatic *Rab18^−/−^* mice (I) were co-stained for β3 tubulin (green, immunostaining) and TRITC-conjugated α-bungarotoxin (red). Large accumulations of microtubules at the NMJ of *Rab18^−/−^* mice were observed. Scale bars: 2 μm (A–D); 10 μm (H,I).

The presence of large accumulations of neurofilaments in motor nerve terminals suggested that normal cytoskeletal organization was disrupted in *Rab18^−/−^* nerves. Therefore, we examined axonal architecture in wild-type and mid-late-symptomatic sciatic nerves using transmission electron microscopy. No abnormalities in myelination were observed in either genotype (Fig. 5A,B,E–G). At higher magnification, gross disorganisation of the cytoskeleton with randomly orientated filaments was observed in sciatic nerves of *Rab18^−/−^* mice (Fig. 5C,D).

Given the cytoskeletal disruption (including disruption of microtubule networks) in *Rab18^−/−^* sciatic nerves, we returned to the NMJ preparations to establish whether microtubule proteins were also included in the abnormal accumulations. Immunohistochemical analysis of the NMJ identified accumulations of β3 tubulin (a marker for neuronal microtubules) at motor nerve terminals (Fig. 5F,G). Overall, it appears that loss of *Rab18* results in the disorganisation of microtubule and neurofilament networks in peripheral nerves.

### Global proteomic profiling of peripheral nerves from *Rab18*-mutant mice reveals molecular defects in cytoskeletal dynamics

In order to identify further molecular alterations that might be associated with the cytoskeletal disorganisation, we used isobaric tags for relative and absolute quantitation (iTRAQ) mass spectrometry proteomics to compare global protein abundance in sciatic nerves from 6-week-old heterozygote (asymptomatic) and *Rab18^−/−^* (symptomatic) mice (*n*=3 per genotype). The screen identified 1337 proteins that were altered in abundance. These were filtered to identify those that had greater than 20% difference in protein levels between the genotypes and were identified by two or more unique peptides. Of the 1337 proteins that were identified, 202 proteins met our stringent filtering criteria – the expression of 112 proteins was increased in *Rab18^−/−^* mice (supplementary material Table S2), and the expression of 90 proteins was decreased (supplementary material Table S3) in *Rab18^−/−^* sciatic nerves.

We validated changes in the abundance of two of these proteins by western blotting extracts from additional sciatic nerves. The 250-kDa neurofilament heavy chain (NEFH) was reduced in abundance in both the iTRAQ and western blotting analysis, whereas the apolipoprotein ApoD was increased in abundance (supplementary material Fig. S3).

In order to ascertain potential systemic consequences of the altered protein levels, pathway analysis was performed using Ingenuity Pathway Analysis (IPA) software. IPA generates functional networks and pathways from the filtered proteomic data based on direct and indirect interactions from published literature. Interestingly, this analysis revealed that a significant number of proteins with altered abundance in *Rab18^−/−^* sciatic nerves were also associated with other neurodegenerative conditions (Table 1). These include movement disorders (44 proteins) and neuromuscular disorders (35 proteins). Importantly, the analysis also revealed clustering of molecular alterations around pathways and processes that are associated with cellular association and organisation (Table 2), including 28 proteins that are known to regulate organisation of the cytoskeleton and 25 proteins that are known to mediate microtubule dynamics. Although individual changes in protein abundance are relatively small, this pathway analysis suggests that RAB18 expression impacts on a variety of candidates and downstream regulators for processes that are involved in cytoskeletal organisation and maintenance. This proteomic profiling of *Rab18^−/−^* sciatic nerve confirmed that RAB18 plays a key role in the cytoskeletal dynamics of the nervous system and identified perturbations in several distinct molecular pathways that are likely to be responsible for maintaining cytoskeletal function.

**Table 1.**
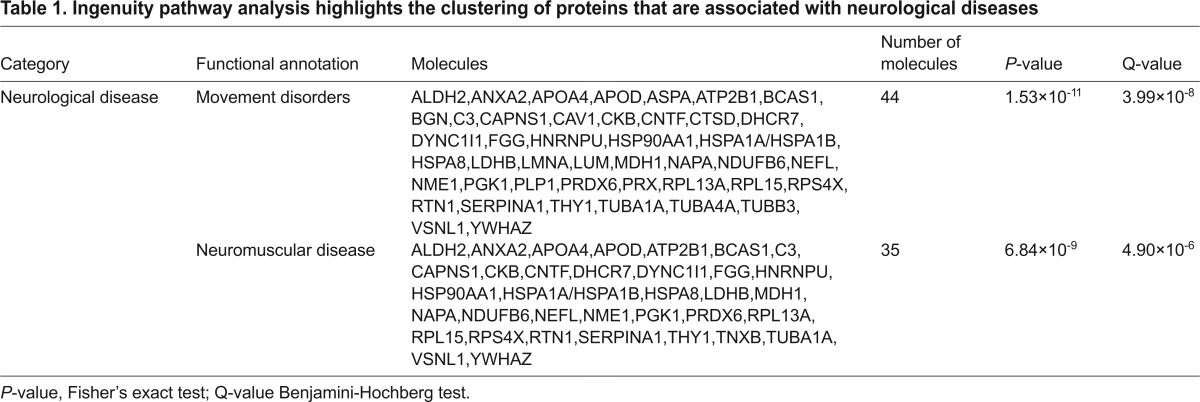
Ingenuity pathway analysis highlights the clustering of proteins that are associated with neurological diseases

**Table 2.**
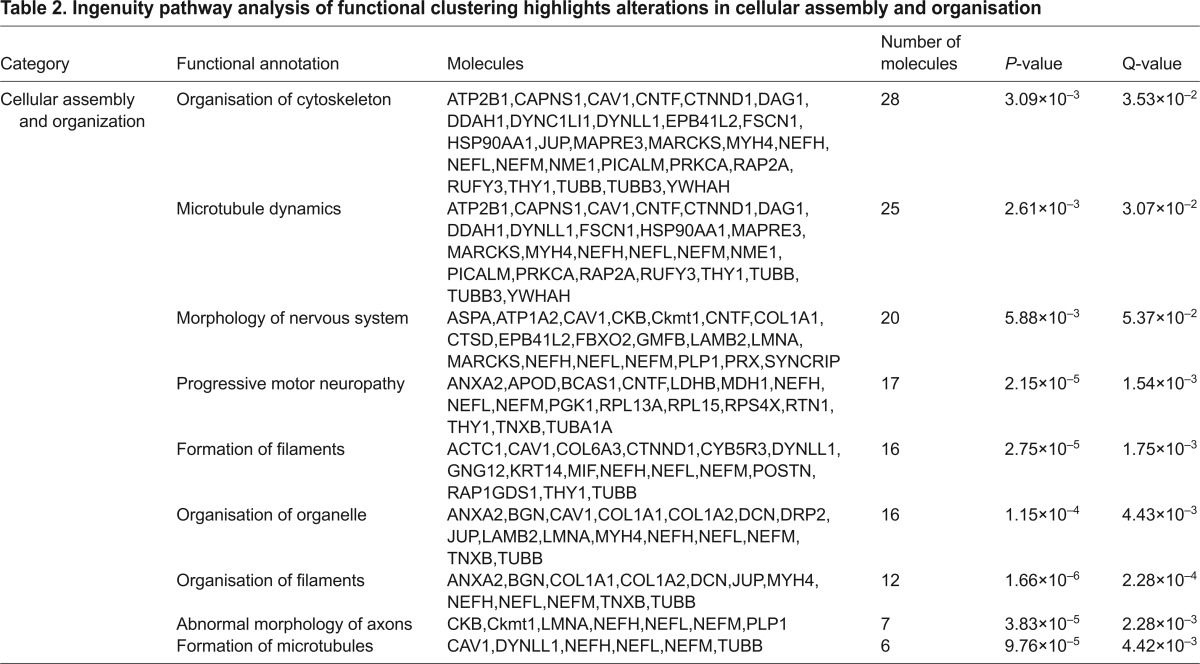
Ingenuity pathway analysis of functional clustering highlights alterations in cellular assembly and organisation

## DISCUSSION

Mutations in *RAB3GAP1*, *RAB3GAP2*, *RAB18* and *TBC1D20* have previously been shown to cause the autosomal recessive disorder WARBM, which is characterised by abnormalities that affect the ocular and neurological systems (Aligianis et al., 2005; Bem et al., 2011; Borck et al., 2011; Liegel et al., 2013). Mouse models for *Rab3gap1* and *Tbc1d20* (*bs*) have been described previously but fail to recapitulate the neurological features of the disorder (Liegel et al., 2013; Sakane et al., 2006). Here, we report the characterisation of a novel *Rab18^−/−^* mouse model that exhibits, with complete penetrance, both the ocular and neurological features that are characteristic of the human disease, including congenital nuclear cataracts, atonic pupils, and hind limb weakness and spasticity. We have shown that loss of *Rab18* does not lead to gross abnormalities in synaptic vesicle recycling at either PNS or CNS synapses but does cause widespread disruption of the neuronal cytoskeleton in peripheral nerves. Proteomic profiling of peripheral nerves revealed perturbations in several molecular pathways that are known to regulate cytoskeletal dynamics in the nervous system and/or to be involved in neurological and neuromuscular disease.

*Rab18^−/−^* mice present at the eye-opening stage with dense nuclear cataracts and atonic pupils. During development of the murine lens, fibre cells terminally differentiate – laying down a complex crystalline substructure that is devoid of nuclei and organelles, thus preventing light scattering and allowing normal lens transparency (Tholozan and Quinlan, 2007). Histological analysis of *Rab18^−/−^* mice identified a delay in closure of the lens vesicle at E12.5 and abnormalities in denucleation of fibre cells in neonates (Fig. 2). Recent studies have highlighted a degree of similarity between neurons and lens fibre cells, which shows that they share a comparable morphology with both having an oriented microtubule system (Frederikse et al., 2012). This suggests there is a link between the cellular pathology that is observed in the peripheral nervous system and in the lens fibre cells in *Rab18^−/−^* mice. Furthermore, a previous study of mice that overexpressed neurofilament light chain reported the development of cataracts, although further characterisation was not undertaken (Monteiro et al., 1990). Individuals with WARBM exhibit a broad array of ocular abnormalities, and the *Rab18^−/−^* mice also displayed atonic pupils, the same pupillary defect that is present in affected individuals.

RAB18 has previously been reported to localise to lipid droplets, where it functions in both lipolysis and lipogenesis (Martin et al., 2005; Ozeki et al., 2005; Pulido et al., 2011). We identified enlarged lipid droplets in *Rab18^−/−^* mouse embryonic fibroblasts, further pointing towards a role for RAB18 in lipid droplet metabolism. Lipid droplet abnormalities have also been reported in other motor neuron diseases. For example, *Spg20^−/−^* mice, modelling Troyer syndrome, show progressive gait defects and increased lipid droplet numbers in adipose tissue (Renvoisé et al., 2012). A reduced lipid droplet diameter has also been reported upon knockdown of atlastin, mutations in which cause hereditary spastic paraplegia, which causes progressive spasticity similar to that observed in WARBM (Klemm et al., 2013). Taken together, these data suggest that neurons are particularly susceptible to abnormalities in lipid droplet homeostasis. Increased lipid droplet volume has also been reported in *bs* MEFs and in fibroblasts taken from individuals with mutations in *RAB3GAP1, TBC1D20* or *RAB18*. However, at this stage, it is unclear whether the lipid droplet phenotype is a cause or consequence of WARBM pathology.

The RAB3GAP1 and RAB3GAP2 proteins have previously been implicated in regulating synaptic vesicle exocytosis through RAB3 (Sakane et al., 2006; Schlüter et al., 2004), and the neurological deficits in WARBM individuals have previously been attributed to a defect in this physiological function. It was therefore surprising that the *Rab18^−/−^* mouse, which recapitulates the affected individual’s neurological deterioration, revealed no gross abnormalities in synaptic vesicle recycling at either CNS or PNS synapses. It is possible that other RAB proteins compensate for the loss of RAB18 in synaptic vesicle recycling, but not for the major neuropathological consequence of widespread cytoskeletal disorganisation in neurons. Our comparison of pathological changes in the motor neurons that innervate muscles in the hind limb versus those that innervate the anterior abdominal wall suggests that neurons with longer axons are more severely affected than those with shorter axons. Moreover, the spatial and temporal nature of cytoskeletal accumulations closely matched the overall progression of weakness that was observed in *Rab18^−/−^* mice, which itself might correlate with the progressive spastic paraplegia that is observed in individuals with WARBM.

The neurofilament accumulations and cytoskeletal disruption that we observed are common pathological features of several other neurodegenerative diseases, including spinal muscular atrophy (SMA) and Charcot Marie Tooth type 2E (CMT2E) (Miller et al., 2002; Perrot and Eyer, 2009). Mouse models of SMA also show presynaptic neurofilament accumulations; however, in these cases, this is associated with progressive loss of motor axons and skeletal muscle denervation (Murray et al., 2008). Additionally, mutations in the neurofilament light gene (NEFL) cause CMT2E, in which axons are enlarged and disorganised neurofilament accumulates (Fabrizi et al., 2004). It is interesting that, despite the observed progression of hind limb weakness in *Rab18^−/−^* mice, the mice did not display any hallmark features of neurodegeneration, such as abnormalities in skeletal muscles, demyelination or ‘dying-back’ pathology at the NMJ. It is tempting to speculate that the mice might exhibit reduced nerve conductivity and axonal transport because of the disruption to the neuronal cytoskeleton that is observed in peripheral nerves. At this stage we cannot exclude the possibility that some CNS pathology underlies the spastic paraplegia that we observed in the *Rab18^−/−^* mice; however, no gross pathology was found upon necropsy of the brain, suggesting that, unlike in the PNS, any abnormalities in the CNS are not gross but might be more subtle at the subcellular level.

Our global proteomic analysis of sciatic nerves from heterozygote and *Rab18^−/−^* littermates confirmed that cytoskeletal disorganisation is a major pathological feature of *Rab18^−/−^* nerve, at both the cellular and molecular level, and revealed significant alterations in the abundance and/or localisation of a range of proteins that are involved in cellular assembly and organisation. This could suggest that there is a defect in trafficking of neurofilaments in *Rab18^−/−^* mice, resulting in accumulations at synaptic terminals and a reduction in levels of it in the sciatic nerve.

In summary, we report here the generation and initial characterisation of a novel *Rab18^−/−^* mouse model that recapitulates many of the characteristic clinical features of WARBM. This has provided us with a foundation in order to begin to understand the pathobiology that underlies the human disease. We have shown that loss of *Rab18* results in specific abnormalities in the development of the eye and the composition and/or stability of the neuronal cytoskeleton. Therapeutic targeting of the molecular pathways found to be altered in the peripheral nerves of *Rab18^−/−^* mice might provide a starting point for the development of novel therapies for WARBM. Furthermore, the pathway analysis identified alterations in the levels of several proteins that are associated with neuromuscular disease and movement disorders, thus indicating that *Rab18^−/−^* mice might be informative in the context of a wide range of related diseases.

## MATERIALS AND METHODS

### Generation of the *Rab18^−/−^* mouse

The *Rab18* mouse [The European Conditional Mouse Mutagenesis Project; strain C57BL/6×129P2/Ola (*Rab18*^Gt(EUCE0233a03)Hmgu^)] was generated as part of the European Mouse Disease Clinic (Eumodic) programme by the Medical Research Council (MRC) Harwell. Details of genetrap and targeting can be found at http://www.informatics.jax.org/javawi2/servlet/WIFetch?page=alleleDetail&key=574626.

### Mouse maintenance

*Rab18* 129P2/Ola and C57BL/6J were maintained as heterozygote breeding pairs in the animal care facilities at the University of Edinburgh under standard conditions. Heterozygote *Rab18^+/−^* mice on a 129P2/Ola background were obtained from MRC Harwell. Most analyses were undertaken on C57BL/6J 129P2/Ola first generation backcrosses. However, mice crossed to ninth generation C57BL/6J showed the same overt phenotype. All animal procedures were performed in accordance with Home Office regulations and institutional guidelines. Litters were genotyped for heterozygote or homozygote presence of the *Rab18* genetrap cassette by standard PCR protocols.

### Reverse transcriptase PCR

RNA was isolated from E11.5 wild-type, heterozygous and *Rab18^−/−^* embryos using the Qiagen RNeasy minikit. cDNA was synthesised from the resultant RNA using Roche First strand cDNA synthesis kit for RT-PCR (AMV) (Roche Applied Science) and the resultant mRNA was amplified using primer pairs directed against *Rab18* exon 1 (Ex1aF, 5′-AGAG-TGGGGTGGGCAAGT-3′) and *Rab18* exon 5 (Ex5R, 5′-CAAAGGTG-TCTCTTCTTGTGACAT-3′); and *Rab18* exon 3 (Ex3F, 5′-AAAACGAT-TTCAGTGGATGGA-3′) and *Rab18* exon7 (Ex7aR, 5′-GGTTCTCACT-TTCCCACAGG-3′). The products were run on a 1% agarose gel.

### Preparation and maintenance of MEFs

MEFs were isolated from E13.5 embryos with the head and organs removed and then minced in Dulbecco’s Modified Eagle’s Medium (DMEM) (Gibco, Invitrogen) that contained 10% foetal calf serum (FCS), 1% penicillin and streptomycin and 3.5μl β-mercaptoethanol (Sigma-Aldrich). The resulting cells were maintained at 37°C under 5% CO_2_ and 3% O_2_.

### Quantitative RT-PCR

Quantitative RT-PCR was performed on cDNA that had been isolated from embryonic fibroblasts using the following probe-primer combinations, which were designed using the Roche Universal Probe Library software. Probe number 33, *Rab18* Forward 5′-GCTGGTCAAGAGAGGTTCAGA-3′ and *Rab18* Reverse 5′-GGTGTCTCTTCTTGTGACATCATAG-3′. Quantitative RT-PCR was performed on a Lightcycler 480 instrument (Roche Applied Science), and TATA box binding protein (*TBP*) was used as a control gene.

### Isolation of crude synaptosomes from brain fractions

Crude synaptosome preparations were generated from the brains of wild-type, heterozygous and *Rab18^−/−^* mice using methods that have been described previously (Wishart et al., 2012).

### Protein extraction and western blot analysis

Protein was extracted from the sciatic nerves of *Rab18^−/−^* mice and the littermate controls. The samples were homogenised in RIPA buffer (Thermo Scientific) containing 1× protease inhibitor cocktail (Roche Applied Science), sonicated three times with 30-second pulses, centrifuged at 16,200 ***g*** at 4°C for 15 minutes and then the supernatant was collected. Protein concentrations were calculated using the Pierce BCA protein assay kit (Thermo Scientific). 25 μg of crude synaptosomes or 10 μg of sciatic nerve samples were separated using Novex 4–12% Bis-Tris pre-cast gels (Life Technologies) and transferred to Hybond nitrocellulose membranes (Hybond ECL, Amersham Biosciences). The membranes were blocked in 4% milk and then incubated with primary rabbit antibody against RAB18 (Eurogentec custom-made antibody, peptide CESENQNKGVKLSHRE), rabbit antibody against RAB3GAP1 (Bethyl Laboratories A310-750A), rabbit antibody against RAB3GAP2 (Abgent, AP9635b) rabbit antibody against RAB3A (Cell Signaling Technology, catalogue number 3930) and rabbit antibody against ApoD (Sigma-Aldrich, catalogue number SAB2700764). A donkey antibody against rabbit IgG and a donkey antibody against mouse IgG conjugated to horseradish peroxidase (Amersham ECL western blotting reagent pack) were used as secondary antibodies. The binding of the antibodies was detected by using the Amersham ECL Plus western blotting detection system.

### Quantitative western blotting Li-COR Odyssey

Protein extraction and electrophoresis was performed as above. The proteins were transferred to PVDF membranes using the Invitrogen I-Blot transfer system for 8.5 minutes. The membranes were incubated in Odyssey blocking buffer for 30 minutes at room temperature before incubation with a primary antibody directed against neurofilament heavy chain (Abcam, catalogue number ab8135) in Odyssey blocking buffer overnight at 4°C. Membranes were then incubated with goat secondary antibody against rabbit IgG (conjugated to IRDye 680 RD) for 90 minutes at room temperature before visualisation and quantification using the Li-COR Odyssey scanner (Li-COR).

### Total protein stain

Protein extraction and electrophoresis was performed as above. The gels were stained with Instant Blue (Expedeon) overnight at room temperature and then washed several times in distilled H_2_O before visualisation. The stained gels were visualised on the Li-COR Odyssey scanner or by using the ImageQuant software.

### Cataract histology

Dissected eyes from neonates and E17.5 embryos were fixed in Davidson’s solution [3 ml 95% ethanol, 2 ml 10% neutral buffered formalin (500 μl formalin and 4.5 ml phosphate buffer), 1 ml glacial acetic acid and 3 ml distilled H_2_O] overnight at 4°C. Embryo heads (E15.5) or intact embryos (E12.5) were fixed in 4% paraformaldehyde (PFA) in PBS overnight at 4°C. All samples were dehydrated through an ascending ethanol series and prepared for paraffin wax embedding. Samples were cut into 7-μm sections and stained using haematoxylin and eosin to analyse the gross anatomy.

### Optical projection tomography

Optical projection tomography was performed on PFA-fixed unpigmented adult eyes, which were mounted in 1% low melting point agarose, dehydrated in methanol and then cleared overnight in BABB (1 part benzyl alcohol, 2 parts benzyl benzoate) as described previously (Sharpe et al., 2002). The eye was then imaged using a Bioptonics optical projection tomography scanner 3001 (Bioptonics, Edinburgh, UK) using tissue autofluorescence (excitation 480 nm, emission 510 nm). The scans were reconstructed using Bioptonics proprietary software and then rendered using Drishti software (Ajay Lamaye, ANU, Canberra, Australia). The movies and stills were created using the same software.

### Examination of ocular reflex

One drop of 1% (w/v) tropicamide (Minims) was dropped on the cornea of *Rab18^−/−^* mice. The mouse was left for 5 minutes in order to allow the pupil to dilate and then the ocular reflex was examined using an indirect ophthalmoscope light.

### Lipid loading of MEFs with oleic acid

Control and *Rab18^−/−^* MEFs were isolated as described above. MEFs were treated with 400 μM oleic acid for 24 hours and labelled with BODIPY 493/503 (Life Technologies) in 150 mM NaCl in H_2_O for 20 minutes at room temperature. Five frames per sample were imaged by using identical microscope settings on a Nikon A1R confocal microscope, and the lipid droplet area was quantified using the ‘Analyse particles’ ImageJ plugin.

### FM1-43fx staining of synaptic vesicle recycling

Mid-late-symptomatic *Rab18^−/−^* mice were killed by cervical dislocation and the muscles were dissected in oxygenated mammalian Ringer solution (120 mM NaCl, 5 mM KCl, 2 mM CaCl_2_, 1 mM MgCl_2_, 0.4 mM NaH_2_PO_4_, 23.8 mM NaHCO_3_ and 5.6 mM D-glucose bubbled with 95% O_2_ and 5% CO_2_). The muscles were stained with bungarotoxin conjugated to TRITC (5 mg/ml, Molecular Probes) for 5 minutes to label postsynaptic acetylcholine receptors and then washed in oxygenated Ringer for 5 minutes. Recycling of synaptic vesicles was then stimulated by using high-K^+^ Ringer solution (75 mM NaCl, 50 mM KCl, 2 mM CaCl_2_, 1 mM MgCl_2_, 0.4 mM NaH_2_PO_4_, 23.8 mM NaHCO_3_ and 5.6 mM D-glucose bubbled with 95% O2 and 5% CO_2_) and fluorescently labelled with the styryl dye FM1-43fx (Molecular Probes, 2.5 μg/ml in high-K^+^ Ringer) for 15 minutes. Following fluorescent labelling, the muscles were washed for 15 minutes in oxygenated Ringer, fixed in 4% PFA (in 1×PBS) for 5 minutes, further washed for 10 minutes in 1×PBS and mounted with Mowiol (Calbiochem).

### Mouse cortical neuron culture

Primary dissociated cortical neuron cultures were prepared from E17.5 *Rab18^−/−^* and wild-type embryos and plated at a density of 1.0×10^7^ cells per coverslip on poly-D-lysine- and laminin-coated coverslips. The cells were maintained in Neurobasal media containing B27, 0.5 mM L-glutamine and 1% (v/v) penicillin and streptomycin (Invitrogen). After 72 hours, the cells were supplemented with 1 μM cytosine β-D-arabinofuranoside to inhibit glial cell proliferation.

### Fluorescent imaging with pHluorin reporter

Fluorescent imaging with sypHy was undertaken as reported previously (Gordon et al., 2011). Cortical neurons were transfected after 7 days *in vitro* (DIV) with 1 μg synaptophysin-pHluorin (sypHy; a gift from Leon Lagnado, LMB Cambridge, Cambridge, UK) by using Lipofectamine 2000, the neurons were imaged after 14–16 DIV. SypHy-transfected cortical cultures were mounted in a Warner imaging chamber with embedded parallel platinum wires (RC-21BRFS) and visualised at 480 nm (>525 nm emission) using a ×40 oil immersion objective on a Zeiss Axio Observer D1 epifluorescence microscope. The cultures were subjected to continuous perfusion with imaging buffer (136 mM NaCl, 2.5 mM KCl, 2 mM CaCl_2_, 1.3 mM MgCl_2_, 10 mM glucose, 10 mM HEPES, pH 7.4) and were stimulated with a train of 300 action potentials that were delivered at 10 Hz (100 mA, 1-millisecond pulse width). Cultures were then subjected to alkaline imaging buffer (as the imaging buffer with 50 mM NH_4_Cl substituted for 50 mM NaCl) to reveal total sypHy fluorescence (total vesicle pool). Fluorescent images were captured at 4-second intervals using a Hamamatsu Orca-ER digital camera (Hamamatsu City, Japan), which were processed offline using ImageJ 1.43 software (National Institutes of Health, USA). Regions of interest of identical size were placed over nerve terminals, and the total fluorescence intensity was monitored over time. All statistical analyses were performed using Microsoft Excel and GraphPad Prism (La Jolla, CA) software. The pHluorin fluorescence change was calculated as FΔ/F0, normalised to either the peak stimulus-evoked fluorescence (to calculate the endocytic time constant – τ) or to the total sypHy fluorescence revealed with alkali buffer (to calculate the peak evoked sypHy response). *n* refers to the number of independent experiments performed.

### Muscle preparation

*Rab18^−/−^* mice and the littermate controls were humanely killed by cervical dislocation. The FDB and deep lumbrical muscles (from the hind paw) and the TVA (from the abdominal wall), were dissected in PBS. The muscles were fixed in 4% PFA in PBS for 15 minutes (mid-late-symptomatic animals) or 10 minutes (early-symptomatic animals), and all muscles were processed for immunohistochemistry.

### Immunohistochemistry

All muscles were permeabilised in 4% Triton X-100 in PBS for 1.5 hours (mid-late symptomatic) or 0.5 hours (early symptomatic) and blocked in 4% bovine serum albumin and 2% Triton X-100 in PBS for 0.5 hours. Muscles were then incubated with primary antibodies directed against 165-kDa neurofilament (2H3 IgG1 supernatant primary, Developmental Studies Hybridoma Bank), SV2 (IgG1 supernatant primary against SV2, Developmental Studies Hybridoma Bank), synaptophysin (DakoCytomation, catalogue number A0010) or β3 tubulin (Abcam, catalogue number ab18207) either over two nights (mid-late symptomatic) or one night (early symptomatic). After 2 hours of washing in PBS, the muscles were incubated for 10 minutes in TRITC-conjugated α-bungarotoxin solution and 2.5 hours in swine Alexa-Fluor-488-conjugated secondary antibody recognising mouse IgG (Dako). Muscles were then mounted in Mowoil (Calbiochem) on glass slides and coverslips.

### Quantification and statistics

All data was collected and analysed using ImageJ and GraphPad Prism 5 software, where mean±s.e.m. Where possible, at least 90 endplates from each muscle preparation, chosen at random, were assessed. For occupancy counts, individual motor neuron endplates were classified as being fully occupied (neurofilament stain entirely covered the endplate), partially occupied (the neurofilament partially occupied the endplate) or vacant (no neurofilament was present in the endplate). To calculate the motor endplate area, images of motor endplates were imported into ImageJ and manually outlined. To calculate the muscle fibre diameter, three diameter measurements from isolated muscle fibres were made using ImageJ, and the mean was then taken. For G-ratio calculations (diameter of the axon divided by the diameter of the axon plus myelin), transmission electron microscopy images were imported into ImageJ, and the area of every intact axon and axon plus myelin was calculated. From this, the diameters were measured and G-ratio calculated. Statistics were calculated using the Mann-Whitney test or unpaired Student’s *t*-test, where appropriate.

### Electron microscopy

Both of the sciatic nerves from *Rab18^−/−^* mice and the littermate controls were dissected in PBS and immediately fixed in 4% PFA with 2.5% glutaraldehyde in PBS for 48 hours at 4°C. The nerves were washed in PBS and incubated in 1% osmium tetroxide for 30 minutes. Following dehydration through an ascending series of ethanol solutions and propylene oxide, the nerves were embedded in Durcupan resin overnight and polymerised for 48 hours at 50°C. Ultrathin sections (~60 nm) were cut and collected on formvar-coated grids (Agar Scientific, UK), stained with uranyl acetate and lead citrate, and then quantitatively assessed in a Philips CM12 transmission electron microscope.

### iTRAQ proteomics

Three of each 6-week-old heterozygote and *Rab18^−/−^* littermate controls were humanely killed by using cervical dislocation. All mice used for this analysis were male, from the same parents and had been backcrossed on to C57BL/6J for nine generations. The sciatic nerve from each leg was dissected in PBS and snap-frozen in liquid nitrogen. Nerves from each genotype were pooled and homogenized in 1:40 (w/v) ratio of 0.5 M triethylammonium bicarbonate pH 8.5, 1% SDS and 1 mM PMSF and then sonicated three times with 30-second pulses and centrifuged at 14,400 r.p.m. at 4°C for 10 minutes. The supernatant was collected for iTRAQ analysis, performed by Dundee Cell Products.

### Proteomic data filtering

Proteomic data was filtered to exclude any protein that was identified by fewer than two unique peptides, and only those of which the abundance changed by greater than 20% were taken forwards for pathway analysis, as previously described (Wishart et al., 2012). It should be noted that six keratin-family members were identified but could not be excluded because they met the required filtering criteria.

## Supplementary Material

Supplementary Material
