# Supplementary material for: A novel mouse model of Warburg Micro syndrome reveals roles for RAB18 in eye development and organisation of the neuronal cytoskeleton
Source: Dis Model Mech. 2014 Apr 24;7(6):711–22. doi: 10.1242/dmm.015222 (PMC4036478; doi:10.1242/dmm.015222)
Supplement: Supplementary Material [file supp_7_6_711__index.html]

A novel mouse model of Warburg Micro syndrome reveals roles for RAB18 in eye development and organisation of the neuronal cytoskeleton — Supplementary Material 

# A novel mouse model of Warburg Micro syndrome reveals roles for RAB18 in eye development and organisation of the neuronal cytoskeleton

## DMM015222 Supplementary Material

**Files in this Data Supplement:**

- **Supplementary Material**
