# Supplementary material for: A novel mouse model of Warburg Micro syndrome reveals roles for RAB18 in eye development and organisation of the neuronal cytoskeleton
Source: Dis Model Mech. 2014 Apr 24;7(6):711–22. doi: 10.1242/dmm.015222 (PMC4036478; doi:10.1242/dmm.015222)
Supplement: Supplementary Material [file supp_7.6.711_DMM015222.pdf]

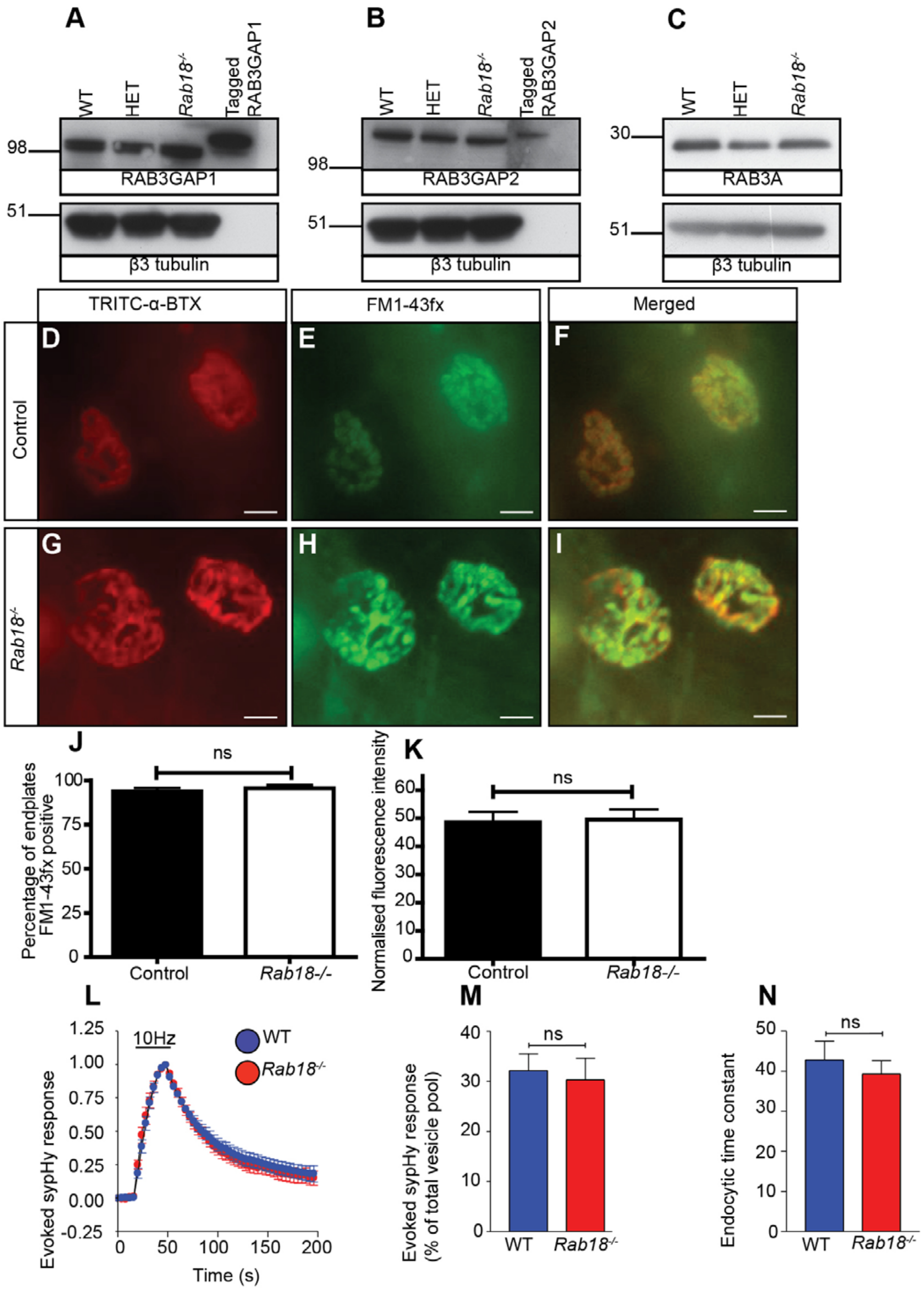

Fig. S1. Loss of RAB18 has no effect on synaptic vesicle recycling in the CNS or PNS. (A-C) Representative western blot analysis of crude synaptosomes from wild type, heterozygote and *Rab18*$^{-/-}$ mice and tagged RAB3GAP1 and RAB3GAP2 (HEK293 transfected MYC tagged RAB3GAP1 and RAB3GAP2 were used to confirm loading) show no alterations in RAB3GAP1 (A), RAB3GAP2 (B) or RAB3A (C) levels. (D-K) Analysis of synaptic vesicle recycling in lumbrical muscles from control (D-F) and mid/late symptomatic *Rab18*$^{-/-}$ (G-I) mice. Endplates were labelled with TRITC-conjugated α-bungarotoxin (D,G) and co-stained with FM1-43fx (E,H) labelling recycling synaptic vesicles. (F and I) are merged images of (D,E) and (G,H) respectively. (I) Bar chart (mean ± SEM) showing the majority of endplates examined effectively took up the FM1-43fx styryl dye irrespective of genotype (*Mann–Whitney test, two tailed, p value=0.3786, n=12 muscles*). (J) Bar chart (mean ± SEM) showing no difference in FM1-43fx fluorescence intensity normalised to TRITC-conjugated α-bungarotoxin in control and *Rab18*$^{-/-}$ littermates *(unpaired two tailed t-test, p value=0.8624, n=10 muscles control and 11 muscles Rab18*$^{-/-}$*).* (L-N) Analysis of synaptic vesicle recycling in cortical neuron cultures from wild type (WT) and *Rab18−/−* (KO) E17.5 embryos. (L) Cortical neuronal cultures were transfected with sypHy and stimulated with a train of 300 action potentials (10 Hz, indicated by bar). Traces illustrate the average fluorescence response over time (wild-type (WT, blue circles, *n*=9) and *Rab18*$^{-/-}$ (KO, red circles, *n*=7) ± SEM). (M,N) Bar graphs illustrate the lack of effect of RAB18 deletion on either (M) the average sypHy peak response (*t*-test *P*=0.74) or (N) the average speed of endocytosis (*t*-test *P*=0.55). *ns=not significant*, scale bar represents 5μm.

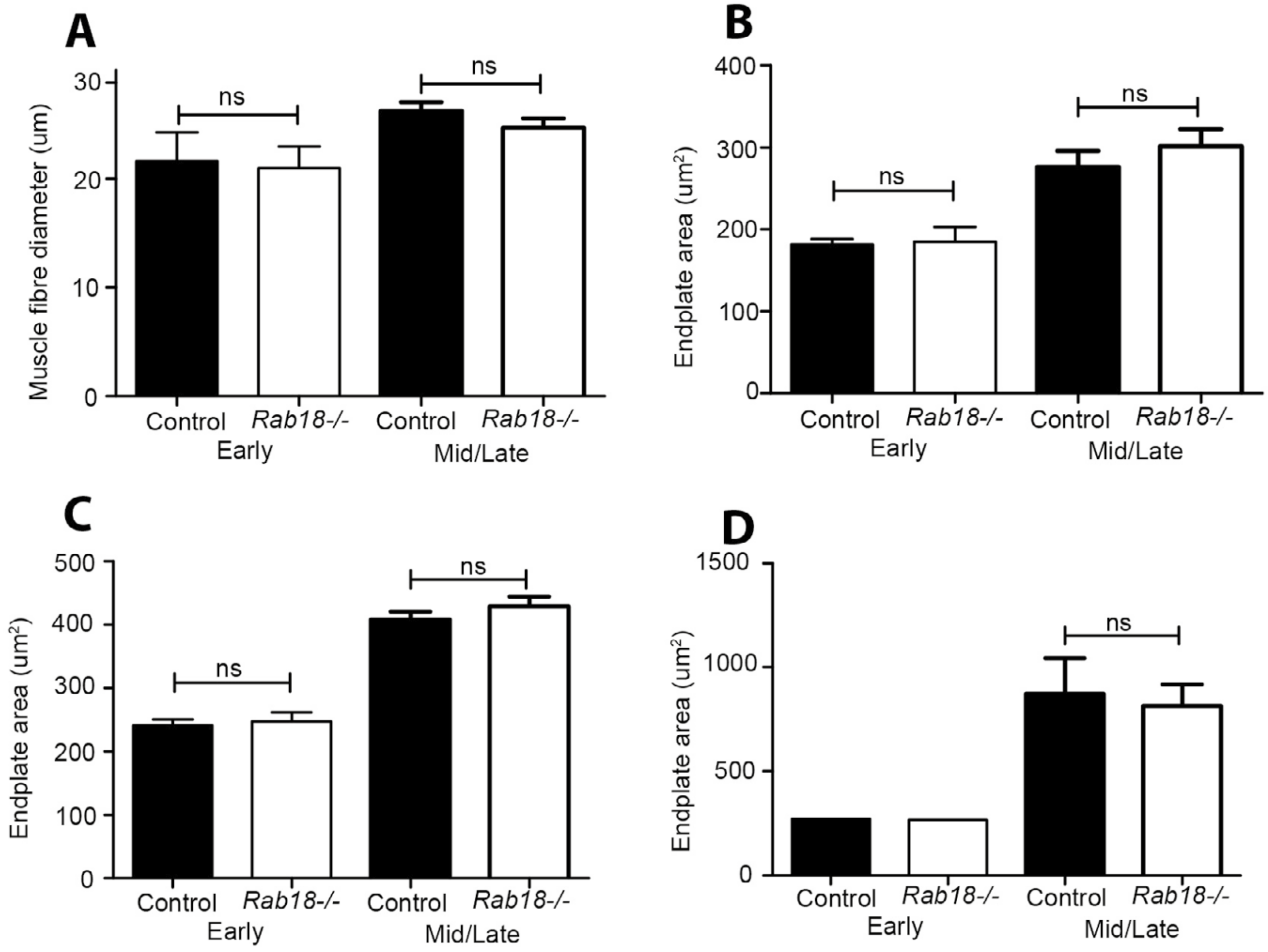

Fig. S2. *Rab18$^{-/-}$* mice show no muscular abnormalities. (A) Bar chart of muscle fibre diameter measurements from whole mount lumbrical muscle preparations. (B-D) Whole mount flexor digitorum brevis (FDB) (B), lumbrical (C) and transverse abdominus (TVA) (D) muscles were stained with TRITC-conjugated α-bungarotoxin and endplate area quantified. No atrophy or swelling of endplates or muscle fibres was observed in early or mid/late symptomatic *Rab18$^{-/-}$* mice. *Mean ±SEM, Unpaired two-tailed t-test. Early symptomatic: FDB p value=0.8661, n=7 muscles, lumbrical p value=0.6966, n=10 muscles, TVA n=1 muscle. Mid/late symptomatic: FDB p value=0.4038, n=9 muscles control and n=8 muscles Rab18$^{-/-}$, lumbrical p value p value=0.2887, n=10 muscles, TVA p value=0.7290, n=3 muscles.*

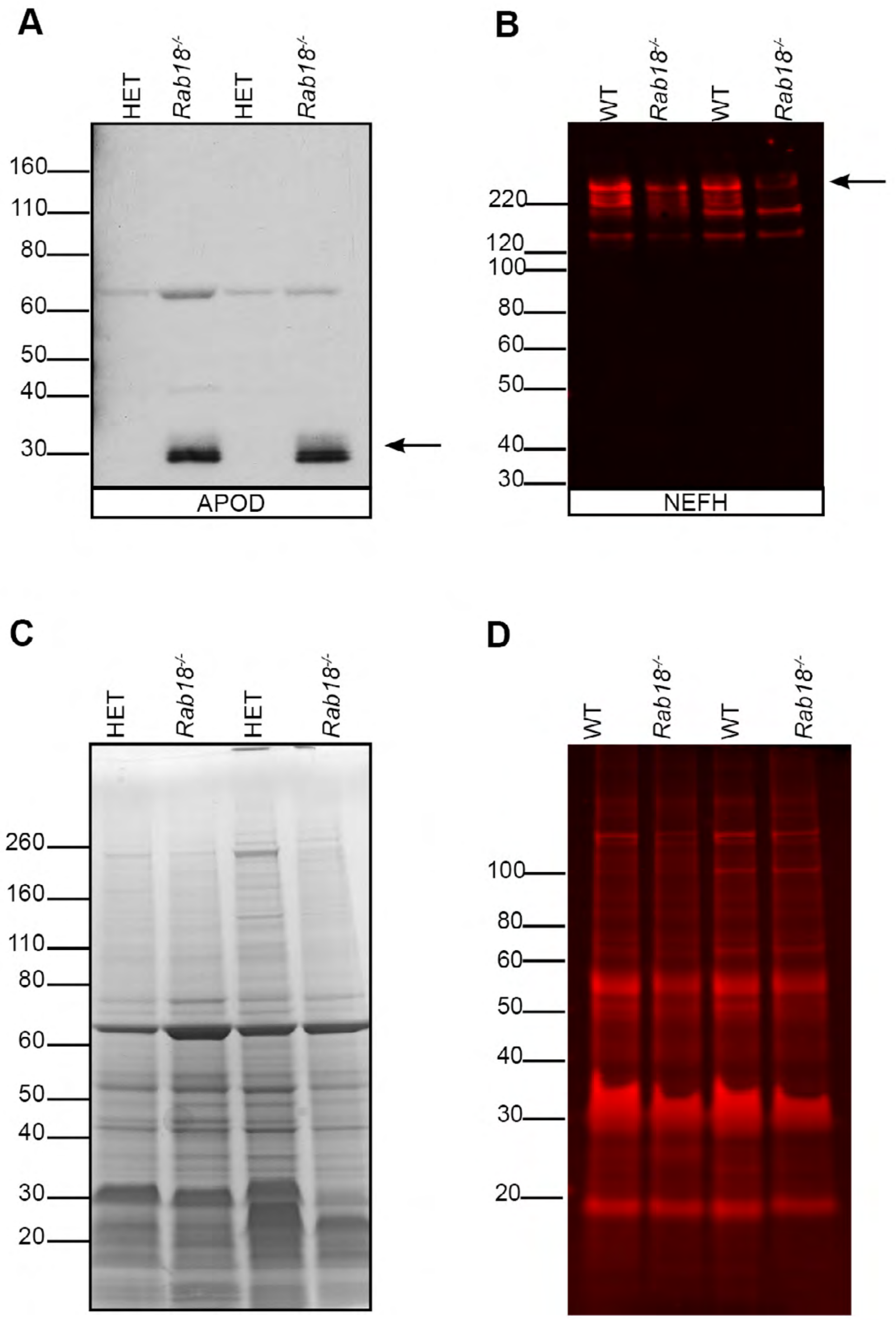

Fig. S3. Proteomic validation in *Rab18*$^{-/-}$ sciatic nerves. (A) Representative Western blot analysis of proteomic validation on heterozygote (HET) and *Rab18*$^{-/-}$ sciatic nerve, showing upregulation of Apolipoprotein D (ApoD) in *Rab18*$^{-/-}$ nerves. (B) Instant Blue stained gel of protein loading using samples run in (A). (C) Representative Western blot analysis of proteomic validation on wild type (WT) and *Rab18*$^{-/-}$ sciatic nerve, showing downregulation of neurofilament heavy chain (NEFH) in *Rab18*$^{-/-}$ nerves. (D) Instant Blue stained gel of protein loading using samples run in (C).

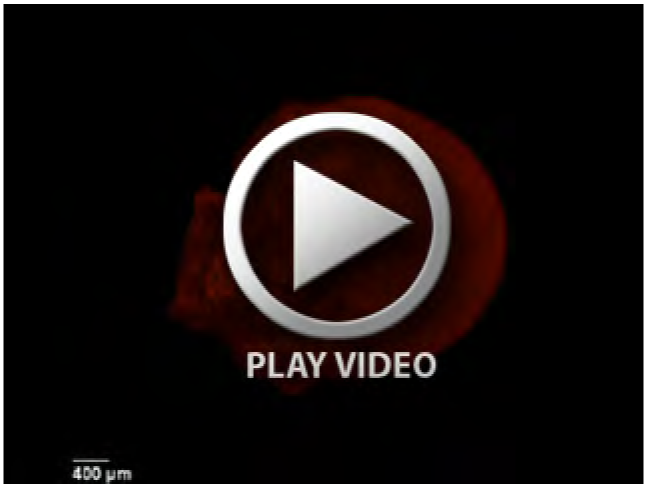

Movie S1. Optical projection tomography on adult wild-type unpigmented eyes. Optical projection tomography on adult wild-type unpigmented eyes showing normal lens.

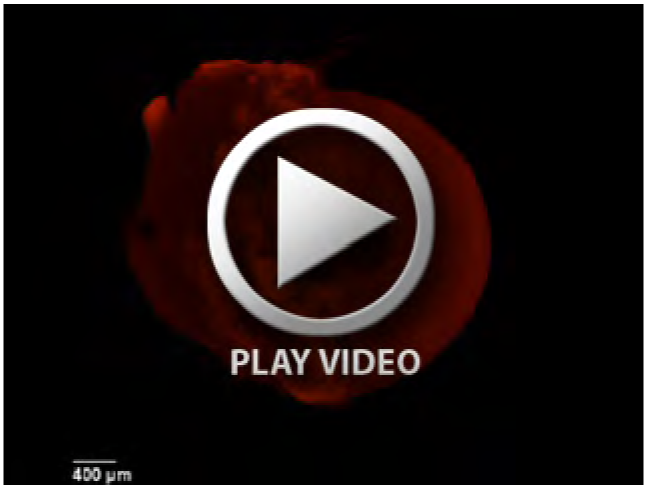

Movie S2. Optical projection tomography on adult *Rab18*$^{-/-}$ unpigmented eyes. Optical projection tomography on adult *Rab18*$^{-/-}$ unpigmented eyes showing dense nuclear cataracts in the centre of the lens.

Table S1. Heterozygote and *Rab18*$^{-/-}$ mice are found at non-Mendelian ratios at weaning but not at various stages of embryogenesis. Ns=not significant

Table S2. iTRAQ identification of proteins >20% upregulated in *Rab18*$^{-/-}$ compared to heterozygotes with 2 or more unique peptides

Table S3. iTRAQ identification of proteins >20% downregulated in *Rab18*$^{-/-}$ compared to heterozygotes with 2 or more unique peptides

Table S1: Heterozygote and *Rab18*$^{-/-}$ mice are found at non-Mendelian ratios at weaning but not at various stages of embryogenesis.
Ns= not significant

Genotype	WT	HET	*Rab18*$^{-/-}$	# litters	Chi-squared
HET x HET litters at weaning	336	388	164	118	p<0.0001
Female BL6 x male HET	188	104		44	p<0.0001
Male BL6 x female HET	38	34		11	Ns
HET x HET embryos	72	133	60	35	Ns

Table S2: iTRAQ identification of proteins >20% upregulated in *Rab18*$^{-/-}$ compared to heterozygotes with 2 or more unique peptides

Symbol	Protein name	Accession number	# Unique peptides	Ratio Rab18/het
MYH4	myosin, heavy chain 4, skeletal muscle	IPI00404837.3	32	4.278
KRT1	keratin 1	IPI00625729.2	2	3.145
KRT5	keratin 5	IPI00470126.4	4	2.842
KRT10	keratin 10	IPI01008564.1	4	2.696
COL1A2	collagen, type I, alpha 2	IPI00988109.1	12	2.556
APOD	apolipoprotein D	IPI00314309.3	5	2.366
KRT13	keratin 13	IPI00136056.1	2	2.355
COL1A1	collagen, type I, alpha 1	IPI00329872.1	9	2.180
KRT14	keratin 14	IPI00227140.1	5	1.928
KRT2	keratin 2	IPI00622240.4	3	1.886
LGALS3	lectin, galactoside-binding, soluble, 3	IPI00989544.2	2	1.871
Ighg	Immunoglobulin heavy chain (gamma polypeptide)	IPI00109910.4	8	1.851
Ighg2a	immunoglobulin heavy constant gamma 2A	IPI00954663.1	2	1.846
ACTC1	actin, alpha, cardiac muscle 1	IPI00114593.1	8	1.731
COL28A1	collagen, type XXVIII, alpha 1	IPI00357842.7	8	1.663
HIST1H1C	histone cluster 1, H1c	IPI00223713.5	4	1.657
IGHM	immunoglobulin heavy constant mu	IPI00468055.3	4	1.653
RPL6	ribosomal protein L6	IPI00313222.5	11	1.645
JUP	junction plakoglobin	IPI00229475.1	2	1.626
HNRNPA2B1	heterogeneous nuclear ribonucleoprotein A2/B1	IPI00405058.6	4	1.549
IGKC	immunoglobulin kappa constant	IPI00850020.1	2	1.545
Pzp	pregnancy zone protein	IPI00624663.3	12	1.543
PCOLCE	procollagen C-endopeptidase enhancer	IPI00120176.1	2	1.533
HNRNPC	heterogeneous nuclear ribonucleoprotein C (C1/C2)	IPI00759870.1	2	1.508
FGA	fibrinogen alpha chain	IPI00115522.3	8	1.493
RPL18A	ribosomal protein L18a	IPI00162790.1	3	1.484

COL6A3	collagen, type VI, alpha 3	IPI00988390.1	2	1.482
COL4A1	collagen, type IV, alpha 1	IPI00109588.4	5	1.466
FGG	fibrinogen gamma chain	IPI00990997.1	12	1.464
RPL15	ribosomal protein L15	IPI00273803.4	2	1.459
FGB	fibrinogen beta chain	IPI00279079.1	8	1.457
RPL13A	ribosomal protein L13a	IPI00223217.6	3	1.414
RPL24	ribosomal protein L24	IPI00880689.1	3	1.409
RPL14	ribosomal protein L14	IPI00133185.3	3	1.408
BCAP31	B-cell receptor-associated protein 31	IPI00230422.6	2	1.408
COL4A2	collagen, type IV, alpha 2	IPI00338452.3	10	1.401
THY1	Thy-1 cell surface antigen	IPI00109727.1	4	1.399
STEAP3	STEAP family member 3, metalloreductase	IPI00830824.1	3	1.371
RPS11	ribosomal protein S11	IPI00762542.2	5	1.362
CAV1	caveolin 1, caveolae protein, 22kDa	IPI00117829.1	3	1.348
TM9SF2	transmembrane 9 superfamily member 2	IPI00988824.1	2	1.343
Rpl10	ribosomal protein L10	IPI00775915.1	5	1.337
LMNA	lamin A/C	IPI00400300.1	32	1.334
NDUFB6	NADH dehydrogenase (ubiquinone) 1 beta subcomplex, 6, 17kDa	IPI00648743.1	2	1.327
Rrbp1	ribosome binding protein 1	IPI00755120.2	3	1.324
LOC638399	60S ribosomal protein L31-like	IPI00677102.1	2	1.323
COPA	coatomer protein complex, subunit alpha	IPI00989407.1	4	1.322
PABPC1	poly(A) binding protein, cytoplasmic 1	IPI00124287.2	2	1.322
RPL7	ribosomal protein L7	IPI00311236.1	10	1.321
MTCH1	mitochondrial carrier 1	IPI00742287.1	2	1.316
Gm5453	predicted gene 5453	IPI00461641.2	3	1.306
RPL18	ribosomal protein L18	IPI00555113.2	5	1.304
HIST1H1B	histone cluster 1, H1b	IPI00230133.5	6	1.304
SEC23A	Sec23 homolog A (S. cerevisiae)	IPI00985577.1	3	1.293
ENTPD2	ectonucleoside triphosphate diphosphohydrolase 2	IPI00115089.2	9	1.293
POSTN	periostin, osteoblast specific factor	IPI00409326.1	10	1.290
GNG12	guanine nucleotide binding protein (G protein), gamma 12	IPI00227838.4	3	1.290
HNRNPL	heterogeneous nuclear ribonucleoprotein L	IPI00985815.1	4	1.289

RPS4X	ribosomal protein S4, X-linked	IPI00990327.1	10	1.281
PICALM	phosphatidylinositol binding clathrin assembly protein	IPI00404434.1	2	1.279
GNB2L1	guanine nucleotide binding protein (G protein), beta polypeptide 2-like 1	IPI00317740.5	2	1.279
APOA1	apolipoprotein A-I	IPI00877236.1	13	1.276
NDUFA9	NADH dehydrogenase (ubiquinone) 1 alpha subcomplex, 9, 39kDa	IPI00120212.2	3	1.272
NSDHL	NAD(P) dependent steroid dehydrogenase-like	IPI00128692.1	5	1.271
CFH	complement factor H	IPI00856390.1	3	1.270
RPS2	ribosomal protein S2	IPI00318492.11	10	1.267
RPS3	ribosomal protein S3	IPI00134599.1	8	1.264
KARS	lysyl-tRNA synthetase	IPI00620145.1	2	1.263
Gm7429	predicted pseudogene 7429	IPI00463886.1	6	1.257
DAD1	defender against cell death 1	IPI00109082.3	2	1.256
MCAM	melanoma cell adhesion molecule	IPI00667748.1	4	1.252
RPL10A	ribosomal protein L10a	IPI00849927.1	5	1.250
DYNC1I1	dynein, cytoplasmic 1, intermediate chain 1	IPI00990353.1	2	1.250
ANXA1	annexin A1	IPI00230395.5	9	1.249
HNRNPU	heterogeneous nuclear ribonucleoprotein U (scaffold attachment factor A)	IPI00970121.1	10	1.247
DDX5	DEAD (Asp-Glu-Ala-Asp) box helicase 5	IPI00420363.3	4	1.246
MGLL	monoglyceride lipase	IPI00953761.1	3	1.245
C3	complement component 3	IPI00323624.4	30	1.244
SYNCRIP	synaptotagmin binding, cytoplasmic RNA interacting protein	IPI01027389.1	2	1.243
LAMB2	laminin, beta 2 (laminin S)	IPI00119065.2	47	1.242
RPS15A	ribosomal protein S15a	IPI00857457.1	2	1.239
KDELR3	KDEL (Lys-Asp-Glu-Leu) endoplasmic reticulum protein retention receptor 3	IPI00320629.3	2	1.238
RPN1	ribophorin I	IPI00309035.2	11	1.238
MT-CO2	cytochrome c oxidase subunit II	IPI00131176.1	3	1.237
RPL8	ribosomal protein L8	IPI00137787.3	6	1.236
TNXB	tenascin XB	IPI00130794.1	14	1.235
DHCR7	7-dehydrocholesterol reductase	IPI00130988.1	3	1.232
ATP2B1	ATPase, Ca++ transporting, plasma membrane 1	IPI00556827.1	3	1.228
P4HB	prolyl 4-hydroxylase, beta polypeptide	IPI00133522.2	8	1.225

RTN1	reticulon 1	IPI00459442.1	3	1.222
RPL9	ribosomal protein L9	IPI00881026.1	3	1.220
CRIP2	cysteine-rich protein 2	IPI00121319.1	3	1.220
EIF4H	eukaryotic translation initiation factor 4H	IPI00222560.7	2	1.219
RPS9	ribosomal protein S9	IPI00420726.3	6	1.218
COPG1	coatomer protein complex, subunit gamma 1	IPI00223437.5	2	1.217
CYB5R3	cytochrome b5 reductase 3	IPI00759904.1	9	1.216
ATP1A2	ATPase, Na+/K+ transporting, alpha 2 polypeptide	IPI00762871.2	14	1.215
MIF	macrophage migration inhibitory factor (glycosylation-inhibiting factor)	IPI00230427.5	2	1.213
CTSD	cathepsin D	IPI00927957.1	5	1.211
RPS16	ribosomal protein S16	IPI00469918.4	4	1.210
PLP1	proteolipid protein 1	IPI00263013.4	8	1.208
RPL29	ribosomal protein L29	IPI00874437.1	2	1.208
MARC2	mitochondrial amidoxime reducing component 2	IPI00123276.1	3	1.207
ITIH2	inter-alpha-trypsin inhibitor heavy chain 2	IPI00970608.1	2	1.202
RUVBL2	RuvB-like 2 (E. coli)	IPI00856697.1	2	1.202

Table S3: iTRAQ identification of proteins >20% downregulated in *Rab18*$^{-/-}$ compared to heterozygotes with 2 or more unique peptides

Symbol	Protein name	Accession #	# Unique peptides	Ratio Rab18/het
ALDH1A1	aldehyde dehydrogenase 1 family, member A1	IPI00626662.3	5	0.503
SERPINA1	serpin peptidase inhibitor, clade A (alpha-1 antiproteinase, antitrypsin), member 1	IPI00123927.1	5	0.539
SDPR	serum deprivation response	IPI00135660.5	3	0.579
VAT1L	vesicle amine transport protein 1 homolog (T. californica)-like	IPI00222759.3	16	0.594
MYL6	myosin, light chain 6, alkali, smooth muscle and non-muscle	IPI00354819.5	2	0.594
AKR1B1	aldo-keto reductase family 1, member B1 (aldose reductase)	IPI00223757.4	13	0.604
PMP2	peripheral myelin protein 2	IPI00553439.2	13	0.605
PRX	periaxin	IPI00762284.1	64	0.616
OGN	osteoglycin	IPI00120848.1	13	0.625
ASPA	aspartoacylase	IPI00881964.1	4	0.626
NEFH	neurofilament, heavy polypeptide	IPI00675855.3	35	0.636
SVIP	small VCP/p97-interacting protein	IPI00460224.3	2	0.639
CNTF	ciliary neurotrophic factor	IPI00125017.1	6	0.641
APOA4	apolipoprotein A-IV	IPI00377351.3	2	0.654
NEFM	neurofilament, medium polypeptide	IPI00323800.6	40	0.657
TCEB2	transcription elongation factor B (SIII), polypeptide 2 (18kDa, elongin B)	IPI00131224.1	2	0.664
FBXO2	F-box protein 2	IPI00153176.2	2	0.664
Serpina3k	serine (or cysteine) peptidase inhibitor, clade A, member 3K	IPI00131830.1	4	0.666
SERPINA1	serpin peptidase inhibitor, clade A (alpha-1 antiproteinase, antitrypsin), member 1	IPI00406302.2	4	0.671
DYNC1LI1	dynein, cytoplasmic 1, light intermediate chain 1	IPI00153421.1	2	0.673
NEFL	neurofilament, light polypeptide	IPI00554928.3	32	0.679
TUBA4A	tubulin, alpha 4a	IPI00117350.1	6	0.680

ANXA5	annexin A5	IPI00317309.5	18	0.683
TFG	TRK-fused gene	IPI00130013.4	7	0.683
HDHD2	haloacid dehalogenase-like hydrolase domain containing 2	IPI00111166.7	2	0.684
DDAH1	dimethylarginine dimethylaminohydrolase 1	IPI00109482.3	11	0.688
PLEKHB1	pleckstrin homology domain containing, family B (evectins) member 1	IPI00858298.1	2	0.690
ANXA6	annexin A6	IPI00554894.3	38	0.693
UBE2K	ubiquitin-conjugating enzyme E2K	IPI00880781.1	2	0.693
COL15A1	collagen, type XV, alpha 1	IPI00648224.1	15	0.702
DCN	decorin	IPI00123196.1	15	0.707
BCAS1	breast carcinoma amplified sequence 1	IPI00330860.3	13	0.708
AARS	alanyl-tRNA synthetase	IPI00321308.4	3	0.708
CKB	creatine kinase, brain	IPI00136703.1	13	0.708
LUM	lumican	IPI00313900.1	13	0.709
LDHB	lactate dehydrogenase B	IPI00229510.5	14	0.710
EPB41L2	erythrocyte membrane protein band 4.1-like 2	IPI00330289.4	32	0.713
TUBA1A	tubulin, alpha 1a	IPI00110753.1	6	0.716
PRKCDBP	protein kinase C, delta binding protein	IPI00126939.1	8	0.716
PTRF	polymerase I and transcript release factor	IPI00117689.1	15	0.721
GLO1	glyoxalase I	IPI00321734.7	6	0.724
MDH1	malate dehydrogenase 1, NAD (soluble)	IPI00336324.11	13	0.727
HSP90AA1	heat shock protein 90kDa alpha (cytosolic), class A member 1	IPI00330804.4	15	0.728
PRKCA	protein kinase C, alpha	IPI00321446.4	2	0.731
NAPA	N-ethylmaleimide-sensitive factor attachment protein, alpha	IPI00118930.1	3	0.732
PRDX6	peroxiredoxin 6	IPI00754071.1	5	0.735
PRELP	proline/arginine-rich end leucine-rich repeat protein	IPI00122293.3	15	0.739
NME1	NME/NM23 nucleoside diphosphate kinase 1	IPI00990246.1	9	0.740
GMFB	glia maturation factor, beta	IPI00467495.4	3	0.741
VSNL1	visinin-like 1	IPI00230418.5	3	0.743
DYNLL1	dynein, light chain, LC8-type 1	IPI00121623.1	2	0.744
STXBP6	syntaxin binding protein 6 (amisyn)	IPI00720103.1	6	0.745
CYB5A	cytochrome b5 type A (microsomal)	IPI00918942.1	3	0.746
TUBB	tubulin, beta class I	IPI00117352.1	4	0.748

HSPA1A/HSPA1B	heat shock 70kDa protein 1A	IPI00798482.20	5	0.748
Ube2n	ubiquitin-conjugating enzyme E2N	IPI00165854.3	4	0.750
TUBB3	tubulin, beta 3 class III	IPI00112251.1	6	0.751
BGN	biglycan	IPI00123194.1	9	0.757
GDI1	GDP dissociation inhibitor 1	IPI00323179.3	10	0.759
MAPRE3	microtubule-associated protein, RP/EB family, member 3	IPI00830432.1	3	0.763
Atp5h	ATP synthase, H+ transporting, mitochondrial F0 complex, subunit d	IPI00881799.1	4	0.764
CACYBP	calcyclin binding protein	IPI00115650.4	2	0.770
USP5	ubiquitin specific peptidase 5 (isopeptidase T)	IPI00881918.1	7	0.771
CTNND1	catenin (cadherin-associated protein), delta 1	IPI00752631.1	3	0.772
RUFY3	RUN and FYVE domain containing 3	IPI00622482.2	10	0.772
YWHAH	tyrosine 3-monooxygenase/tryptophan 5-monooxygenase activation protein, eta polypeptide	IPI00227392.5	5	0.773
SERPINA1	serpin peptidase inhibitor, clade A (alpha-1 antiproteinase, antitrypsin), member 1	IPI00129755.2	3	0.774
Ckmt1	creatine kinase, mitochondrial 1, ubiquitous	IPI00128296.1	7	0.777
PGK1	phosphoglycerate kinase 1	IPI00555069.3	17	0.777
DRP2	dystrophin related protein 2	IPI00830746.1	14	0.779
WBP2	WW domain binding protein 2	IPI00648905.1	3	0.780
ANXA3	annexin A3	IPI00132722.9	6	0.782
HSPA4L	heat shock 70kDa protein 4-like	IPI00317711.1	8	0.786
HSPA8	heat shock 70kDa protein 8	IPI00323357.3	25	0.786
YWHAZ	tyrosine 3-monooxygenase/tryptophan 5-monooxygenase activation protein, zeta polypeptide	IPI00116498.1	11	0.786
SNX6	sorting nexin 6	IPI00111827.5	2	0.789
DAG1	dystroglycan 1 (dystrophin-associated glycoprotein 1)	IPI00122273.1	7	0.790
ANXA2	annexin A2	IPI00468203.3	25	0.790
GSTM3	glutathione S-transferase mu 3 (brain)	IPI00990550.1	3	0.791
IDH1	isocitrate dehydrogenase 1 (NADP+), soluble	IPI00762452.2	11	0.791
RAP2A	RAP2A, member of RAS oncogene family	IPI00396701.3	2	0.795
IDH3A	isocitrate dehydrogenase 3 (NAD+) alpha	IPI00608078.1	3	0.796
MARCKS	myristoylated alanine-rich protein kinase C substrate	IPI00229534.5	4	0.798
RAP1GDS1	RAP1, GTP-GDP dissociation stimulator 1	IPI00653794.2	21	0.798

PPP1R7	protein phosphatase 1, regulatory subunit 7	IPI00129319.3	2	0.799
CAPNS1	calpain, small subunit 1	IPI00830335.1	3	0.799
FSCN1	fascin homolog 1, actin-bundling protein (Strongylocentrotus purpuratus)	IPI00353563.4	16	0.799
PYGB	phosphorylase, glycogen; brain	IPI00229796.3	20	0.799
ALDH2	aldehyde dehydrogenase 2 family (mitochondrial)	IPI00111218.1	13	0.799
